# International Observational Analysis of Evolution and Outcomes of Chronic Stable Angina: The Multinational CLARIFY Study

**DOI:** 10.1161/CIRCULATIONAHA.121.054567

**Published:** 2021-07-15

**Authors:** Jules Mesnier, Gregory Ducrocq, Nicolas Danchin, Roberto Ferrari, Ian Ford, Jean-Claude Tardif, Michal Tendera, Kim M. Fox, Philippe Gabriel Steg

**Affiliations:** 1 Assistance Publique-Hôpitaux de Paris, Hôpital Bichat, France (J.M., G.D., P.G.S.).; 2 French Alliance for Cardiovascular Trials, Paris (J.M., G.D., P.G.S.).; 3 Institut national de la santé et de la recherche médicale U1148, Paris, France (G.D., P.G.S.).; 4 Université de Paris, France (G.D., N.D., P.G.S.).; 5 Assistance Publique-Hôpitaux de Paris, Hôpital Européen Georges Pompidou, France (N.D.).; 6 Maria Cecilia Hospital, Gruppo Villa Maria Care & Research, Cotignola (Ravenna), Italy (R.F.).; 7 Centro Cardiologico Universitario di Ferrara, University of Ferrara, Italy (R.F.).; 8 Robertson Centre for Biostatistics, Glasgow, United Kingdom (I.F.).; 9 Montreal Heart Institute, University of Montreal, Canada (J.-C.T.).; 10 School of Medicine in Katowice, Medical University of Silesia, Poland (M.T.).; 11 Imperial College, Royal Brompton Hospital, London, United Kingdom (K.M.F., P.G.S.).

**Keywords:** angina pectoris, stable angina, stable coronary artery, disease

## Abstract

**Background:**

Although angina is common in patients with stable coronary artery disease, limited data are available on its prevalence, natural evolution, and outcomes in the era of effective cardiovascular drugs and widespread use of coronary revascularization.

**Methods:**

Using data from 32 691 patients with stable coronary artery disease from the prospective observational CLARIFY registry (Prospective Observational Longitudinal Registry of Patients with Stable Coronary Artery Disease), anginal status was mapped each year in patients without new coronary revascularization or new myocardial infarction. The use of medical interventions in the year preceding angina resolution was explored. The effect of 1-year changes in angina status on 5-year outcomes was analyzed using multivariable analysis.

**Results:**

Among 7212 (22.1%) patients who reported angina at baseline, angina disappeared (without coronary revascularization) in 39.6% at 1 year, with further annual decreases. In patients without angina at baseline, 2.0% to 4.8% developed angina each year. During 5-year follow-up, angina was controlled in 7773 patients, in whom resolution of angina was obtained with increased use of antianginal treatment in 11.1%, with coronary revascularization in 4.5%, and without any changes in medication or revascularization in 84.4%. Compared to patients without angina at baseline and 1 year, persistence of angina and occurrence of angina at 1 year with conservative management were each independently associated with higher rates of cardiovascular death or myocardial infarction (adjusted hazard ratio, 1.32 [95% CI, 1.12−1.55] for persistence of angina; adjusted hazard ratio, 1.37 [95% CI, 1.11−1.70] for occurrence of angina) at 5 years. Patients whose angina had resolved at 1 year with conservative management were not at higher risk of cardiovascular death or myocardial infarction than those who never experienced angina (adjusted hazard ratio, 0.97 [95% CI, 0.82−1.15]).

**Conclusions:**

Angina affects almost one-quarter of patients with stable coronary artery disease but resolves without events or coronary revascularization in most patients. Resolution of angina within 1 year with conservative management predicted outcomes similar to lack of angina, whereas persistence or occurrence was associated with worse outcomes. Because most patients with angina are likely to experience resolution of symptoms, and because there is no demonstrated outcome benefit to routine revascularization, this study emphasizes the value of conservative management of stable coronary artery disease.

**Registration:**

URL: https://www.isrctn.com; Unique identifier: ISRCTN43070564.

## Abstract 2

Supplemental Digital Content is available in the text.

**Clinical Perspective**

**What Is New?**

- Angina affects almost one-quarter of patients with stable coronary artery disease but resolves with conservative management and without cardiovascular events in most patients.
- In this medically well-treated population, angina largely resolves without changes in medication or revascularization.
- Resolution of angina within 1 year with conservative management predicted outcomes similar to absence of angina, whereas persistence or occurrence was associated with poor cardiovascular outcomes.

**What Are the Clinical Implications?**

- Given that the benefit of antianginal medications and revascularization is restricted to symptom improvement in stable coronary artery disease, this study suggests that conservative management is an effective strategy for patients with stable angina.
- The low rate of cardiovascular events and favorable evolution of angina in most patients allow for a watchful waiting strategy before failure of medical management is declared.
- The frequency of resolution of angina in the first year suggests that trials testing antianginal drugs may need to incorporate a longer run-in period.

**Editorial, see p 524**

Angina pectoris is a common manifestation of stable coronary artery disease (CAD) and negatively affects quality of life.^1–3^ Lifestyle changes, pharmacological treatment, and coronary revascularization can improve symptoms.^4–10^ However, in most randomized trials exploring antianginal strategies or drugs in stable CAD, the burden of angina improved over time in the control group.^6,8,11–14^ Although this improvement could be a result of optimization of medical treatments and cardiovascular risk factors during the trial, it could also reflect the natural history of the condition.^15,16^

Angina pectoris is also associated with poor outcomes.^2,3,17–19^ However, antianginal treatments have not been proven to improve outcomes in chronic stable angina.^20,21^ Even β-blockers, largely used on the basis of a mortality reduction after myocardial infarction, have not been associated with improved outcomes in stable angina.^22,23^ Likewise, invasive management with a view to coronary revascularization does not reduce the risk of death or myocardial infarction in patients with stable angina or myocardial ischemia, although it improves symptoms.^13,14,24,25^

In the era of effective antianginals treatments, evidence-based secondary prevention therapy, and widespread use of coronary revascularization, we sought to describe the prevalence and time course of angina in patients with stable CAD as well as the effect of changes in anginal status on outcomes.

## Methods

The data, analytic methods, and study materials will be made available to other researchers for purposes of reproducing the results or replicating the procedure, subject to request.

The CLARIFY registry (Prospective Observational Longitudinal Registry of Patients with Stable Coronary Artery Disease) has been described.^26^ It included 32 703 outpatients with stable CAD enrolled in 45 countries (Table I in the Data Supplement) between November 26, 2009, and June 30, 2010.

Stable CAD was defined as the presence of at least 1 of the following: documented myocardial infarction >3 months before enrollment, angiographic demonstration of >50% coronary stenosis, chest pain with evidence of myocardial ischemia, and history of coronary artery bypass grafting or percutaneous coronary intervention >3 months before enrollment. The distribution of patients according to each inclusion criterion is shown in Figure I in the Data Supplement.

Exclusion criteria were hospital admission for cardiovascular reasons (including coronary revascularization) in the past 3 months, planned revascularization or conditions compromising the participation for the 5 years of planned follow-up, including advanced heart failure or severe valve disease, and history of valve repair or replacement.

The registry was observational, did not interfere with medical management, and reflects routine practice. In each site, patients were recruited over a brief period to achieve near-consecutive enrollment, and data were prospectively collected by investigators on dedicated electronic forms.

The study was done in accordance with the Declaration of Helsinki, and local ethical approval was obtained in all countries. All patients gave written informed consent. This analysis is restricted to patients with available baseline angina status, and corresponds to a prespecified population of interest in the CLARIFY registry.^26^

Investigators completed standardized electronic case report forms at baseline and every year (plus or minus 3 months) during patients’ visits, for up to 5 years. Patients were censored after the 5-year visit or at 5 years plus 3 months after inclusion. Each year, symptoms, clinical examination, biological results, treatments, and outcomes were recorded. Outcomes were not adjudicated, but investigators were provided with clear definitions for each outcome. To ensure data quality, each year, 1% of sites were randomly selected for onsite audit of 100% of the data, and case reports forms were centrally monitored for completeness and consistency. All events were verified at the source during the audits.

The presence of angina was recorded at baseline and yearly visits, and was defined as chest pain during physical exertion or equivalent symptoms necessitating occasional or permanent use of antianginal drugs in the judgment of the investigator. We aimed to describe the natural history of angina pectoris. Patients were categorized according to the presence of angina at baseline. Yearly changes in angina status are presented, up to the first myocardial infarction or coronary revascularization, given that these events can result in complications that may affect anginal status. Hence, patients were censored after occurrence of myocardial infarction or revascularization. Conversely, data from patients experiencing unstable angina that did not lead to coronary revascularization or myocardial infarction were analyzed.

To assess the consequences of angina evolution on cardiovascular outcomes, patients were categorized into 4 groups according to the evolution of angina between baseline and 1 year: persistence, resolution, occurrence (if angina was absent at baseline and appeared at 1 year), or absence of angina (if angina was absent at baseline and 1 year).

In patients with regression of angina (if angina was present at year N and absent at year N+1), control of angina was ascribed to coronary revascularization if the latter had been performed during the previous year as an elective procedure or for unstable angina. If no coronary revascularization had been performed, control of angina was ascribed to medical therapy if antianginal treatments, including β-blockers, ivabradine, calcium antagonists, long-acting nitrates, or other antianginal drugs (trimetazine, ranolazine, nicorandil, and molsidomine) had been added or increased and if β-blockers were switched or dosage increased. In patients who had not undergone revascularization or changes in antianginal therapy in the year before resolution, the control of angina was deemed as being without new medical intervention.

Subgroup analyses were performed in patients with previous myocardial infarction at baseline, known ischemia at baseline, diabetes, and multivessel CAD.^3^

The primary outcome was the composite of cardiovascular death or nonfatal myocardial infarction. Secondary outcomes were all-cause death, cardiovascular death, fatal or nonfatal myocardial infarction, and elective myocardial revascularization (percutaneous coronary intervention or coronary artery bypass grafting).

### Statistical Analysis

The evolution of angina was plotted using Sankey plots (http://sankeymatic.com). To map the evolution of angina, the last known value of angina during follow-up was imputed when no value was available. A sensitivity analysis was performed without imputation. No imputation was used in survival analyses in which patients with missing angina status at 1 year were censored.

Continuous variables were compared using the χ^2^ test and continuous variables using analysis of variance or the Wilcoxon rank-sum test, according to the distribution. Because 2 comparisons of baseline characteristics according to the evolution of angina at 1 year were performed, a *P* value threshold of <0.025 was used for the descriptive analysis, after applying Bonferroni correction. A multivariable Cox proportional hazards model was used to assess the association between the evolution of angina and cardiovascular outcomes. Selection of variables for the multivariable model was based on clinical importance and a previous analysis from the CLARIFY registry,^3^ and included age; geographic region; sex; baseline smoking status; dyslipidemia; family history of premature CAD; hypertension; diabetes; physical activity; peripheral artery disease; previous myocardial infarction, percutaneous coronary intervention, coronary artery bypass graft, stroke, or transient ischemic attack, or hospitalization for heart failure; atrial fibrillation or flutter; asthma or chronic obstructive pulmonary disease; body mass index; systolic and diastolic blood pressure at baseline; heart rate; and heart failure symptoms at baseline. Statistical analyses were performed using Python 3.0 (Pandas, Sci-Py and Lifelines packages).

## Results

Among 32 703 patients in CLARIFY, 32 691 had information on baseline anginal status with a median follow-up of 5.0 years (quartiles 1−3: 4.8−5.1). Overall, their mean age was 64.2±10.5 years, 71.0% had treated hypertension, 29.0% diabetes, 59.9% previous myocardial infarction, 58.6% previous percutaneous coronary intervention, and 23.6% coronary artery bypass graft. High use of secondary prevention drugs was reported, with 92.3% reporting lipid-lowering drugs, 95.2% at least 1 antiplatelet agent, 75.3% a β-blocker, and 76.2% renin-angiotensin system inhibitors. Overall, 7212 (22.1%) reported angina. Clinical characteristics according to angina status are described in Table 1.^3^ The prevalence of angina according to each inclusion criterion is presented in Table II in the Data Supplement. The primary outcome occurred in 9.1% of patients with angina at baseline versus 6.5% without (*P*<0.001). Angina at baseline was an independent predictor of the primary outcome (adjusted hazard ratio [HR], 1.20, [95% CI, 1.08−1.34]; Table III in the Data Supplement).

**Table 1.**
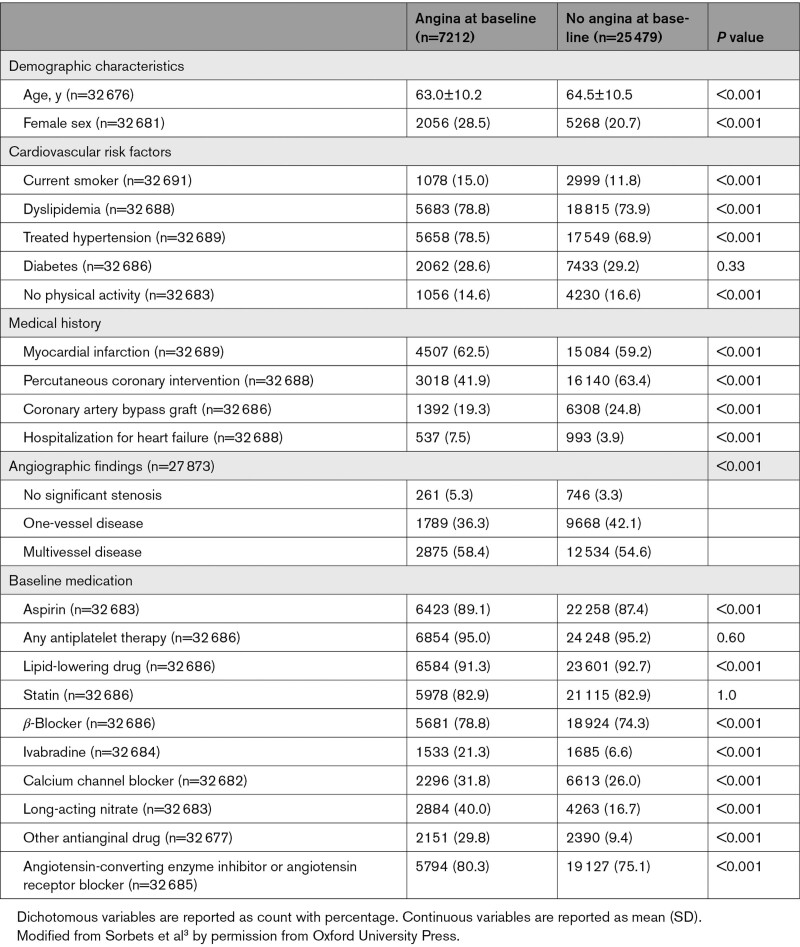
Baseline Characteristics According to Angina Status at Inclusion

**Figure 1.**
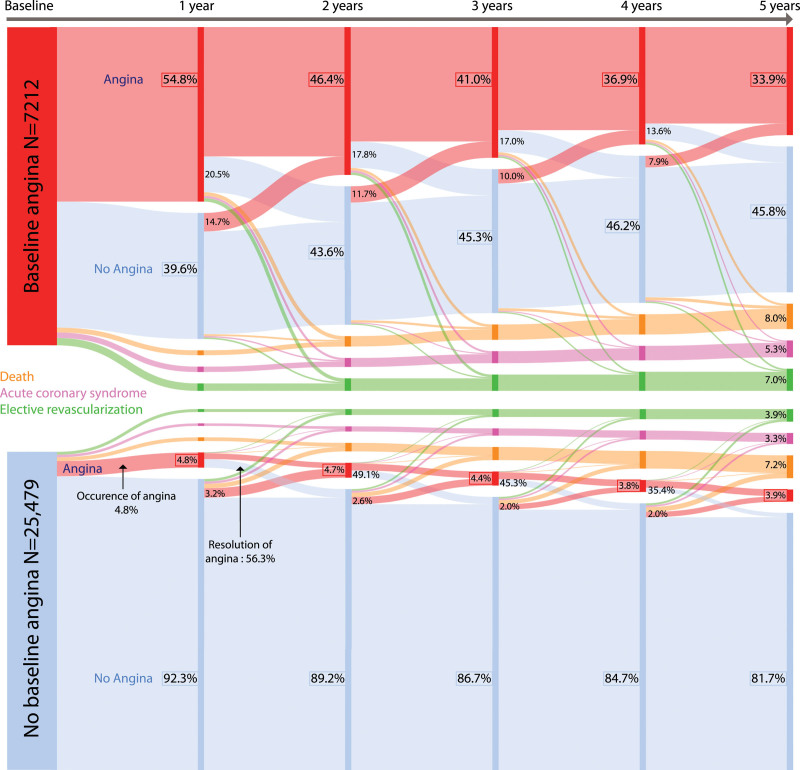
**Evolution of angina in patients with stable coronary artery disease.** Percentages on nodes reflect the proportion of patients compared with the initial group (baseline or no baseline angina). Percentages shown on the crossover represent the proportion of patients according to the value of origin node. Missing angina status values were imputed using the last known value.

Among patients with angina at baseline, angina disappeared (without coronary revascularization) in 39.6% between baseline and year 1, with further decreases annually (Figure 1). At 5 years, 33.9% of patients with angina at baseline still experienced anginal symptoms, 8.0% had died, 5.3% had had a myocardial infarction or undergone urgent revascularization, 7.0% had undergone elective revascularization, and 45.8% were event-free and angina-free.

In the 25 479 patients without angina at baseline, 2.0% to 4.8% developed angina annually. At the end of follow-up, only 3.9% of patients had anginal symptoms, 7.2% had died, 3.9% had undergone elective revascularization, and 3.3% had had either a myocardial infarction or urgent revascularization. Overall, 81.7% of the patients without angina at baseline were event-free and angina-free at 5 years.

Anginal status was missing in 5.3% of patients at 1-year follow-up and in 27.3% at 5-year follow-up; characteristics of these patients are shown in Table IV in the Data Supplement. A sensitivity analysis without imputation of missing values for anginal status yielded similar results to those of the main analysis (Figure II in the Data Supplement). A Sankey plot at scale of the population is available in Figure III in the Data Supplement.

There were 7773 patients in whom angina regressed at any follow-up visit. Table 2 shows the interventions administered in the year angina resolved. Overall, angina was controlled by coronary revascularization (elective or for unstable angina) in 4.5% of patients, increases or changes in antianginal medications in 11.1%, and regressed without new medical intervention in 84.4% of cases. Angina control with medications was achieved by adding at least 1 antianginal drug in 46.9%, switching treatments in 40.0%, and increasing β-blocker dose in 13.0% of patients. Of note, among patients with angina at baseline who underwent (either elective or for unstable angina) revascularization during year 1 (n=224), percutaneous coronary intervention was used in 70.1% and coronary artery bypass grafting in 29.9%, and the frequency of angina resolution at 1 year was greater after surgery than after percutaneous coronary intervention (74.6% versus 44.6%, respectively; *P*<0.001).

**Table 2.**
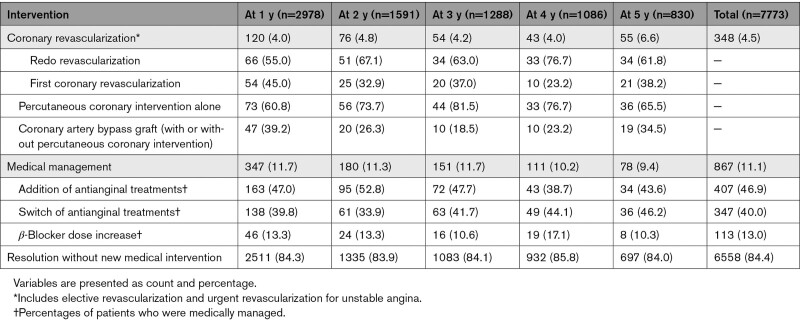
Medical Intervention During the Year Angina Symptoms Regressed

Baseline characteristics, symptoms, and treatments according to the evolution of angina between baseline and 1 year are shown in Tables 3 and 4. In the overall population, at 1 year, 408 (1.2%) patients had died and 728 (2.2%) had either had a myocardial infarction or undergone coronary revascularization. In the remaining patients, angina was persistent in 3660 (11.2%), regressed (without coronary revascularization) in 2858 (8.7%), and occurred in 1216 (3.7%); 22 106 (67.6%) patients had no anginal symptoms and 1715 (5.2%) had no information on angina at 1 year. The characteristics of patients with missing values at 1 and 5 years are shown and compared with patients with follow-up in Table IV in the Data Supplement.

**Table 3.**
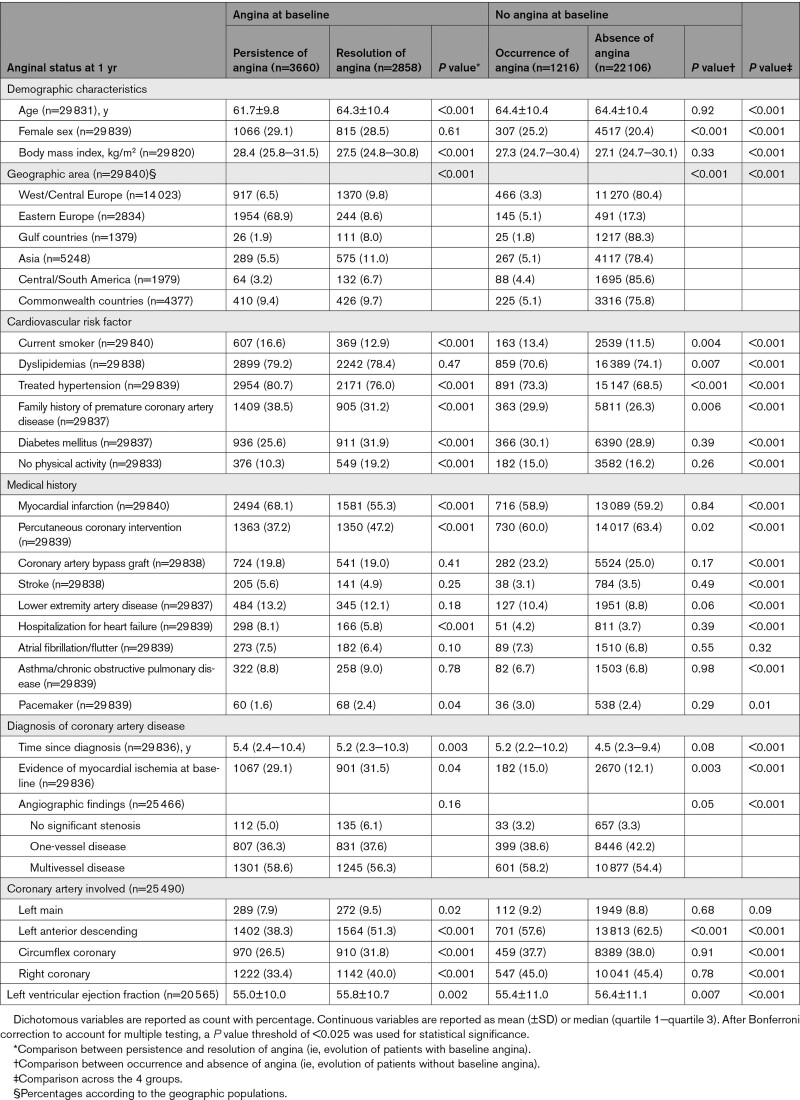
Baseline Characteristics According to the Evolution of Angina at 1 Year

**Table 4.**
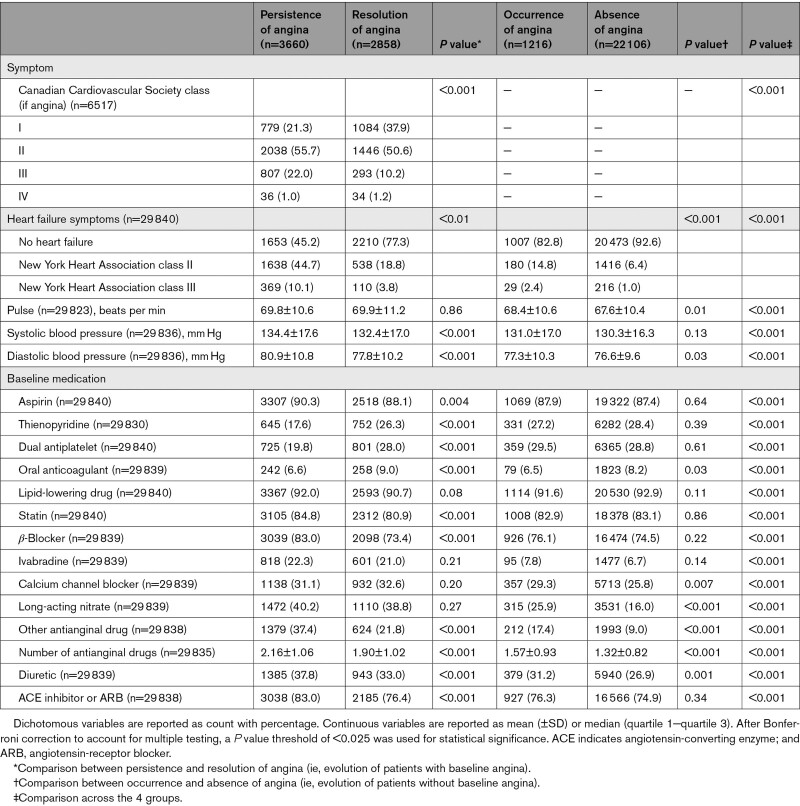
Baseline Symptoms and Treatments According to the Evolution of Angina at 1 Year

Patients with resolution of angina at 1 year were more likely to be older and have lower blood pressure but higher rates of diabetes and no physical activity compared to patients with persistence of angina (Tables 3 and 4). They were also less likely to have previous myocardial infarction and had higher rates of previous percutaneous coronary intervention.

The severity of angina at baseline, 1-year, and 5-year follow-up is described in Table V in the Data Supplement. Patients in whom angina resolved at 1 year had a less severe angina class at baseline than patients in whom angina persisted (*P*<0.001). Likewise, they used fewer nitroglycerin puffs per month (*P*<0.001) and had fewer angina attacks per month (*P*<0.001). In addition, the severity of angina, as measured from the number of nitroglycerin puffs/months, as well as the number of angina attacks per months, appeared to decline over time.

In patients without angina at baseline, angina occurred in 4.8% at 1 year. These patients were more frequently women, with more cardiovascular risk factors (Table 3). They were less likely to have had a previous percutaneous coronary intervention.

**Figure 2.**
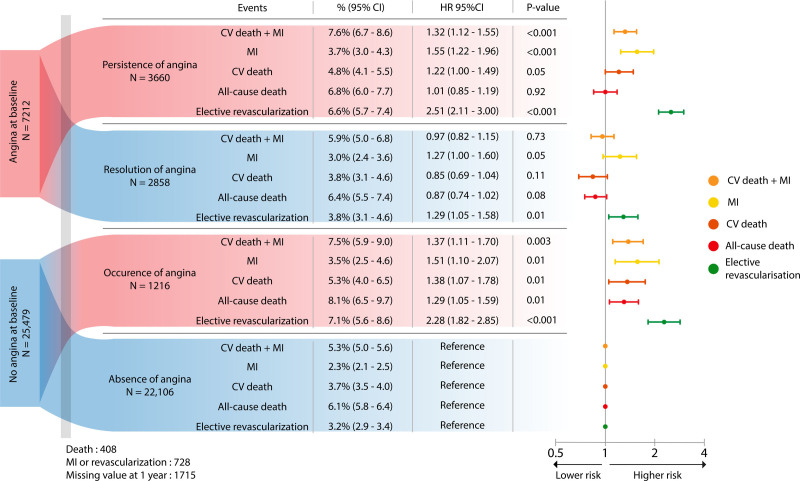
**Clinical outcomes according to the evolution of angina at 1 year.** HRs were adjusted and estimated from a multivariable Cox proportional hazards model. CI indicates confidence interval; CV, cardiovascular; HR, hazard ratio; and MI, myocardial infarction.

The association between changes in anginal status at 1 year and subsequent 5-year outcomes is presented in Figure 2. Compared with patients who did not experience angina at baseline and 1 year, persistence and occurrence of angina were both associated with worse cardiovascular outcomes. Persistence of angina was associated with higher rates of the composite primary outcome (adjusted HR; 1.32 [95% CI, 1.12−1.55]) and of each individual component except all-cause death. Occurrence of angina was associated with higher rates of cardiovascular death and myocardial infarction (adjusted HR, 1.37 [95% CI, 1.11−1.70]), each individual component, and all-cause death (adjusted HR, 1.29 [95% CI, 1.05−1.59]). Patients who had resolution of angina did not experience higher rates of cardiovascular death or myocardial infarction, but had a higher rate of myocardial infarction (adjusted HR, 1.27 [95% CI, 1.00−1.60]). Patients who experienced angina at either baseline or 1 year, compared with patients who did not, had higher rates of elective coronary revascularization.

Patients in Eastern Europe experienced a higher prevalence of angina symptoms compared with the rest of the cohort (78.1% versus 16.3%). We therefore performed a sensitivity analysis excluding patients from Eastern Europe. This yielded similar results about the impact of angina and its evolution on outcomes (Figure IV in the Data Supplement). In areas of high and low prevalence of angina, angina at baseline was associated with the primary outcome (adjusted HR, 1.49 [95% CI, 1.03–2.14]; *P*=0.03 and adjusted HR, 1.17 [95% CI, 1.05–1.31]; *P*=0.005 in Eastern Europe and the rest of the world, respectively). We assessed the relationship between the evolution of angina and subsequent prognosis when excluding Eastern European patients and found results similar to those of the primary analysis (Figure V in the Data Supplement).

Figure VI in the Data Supplement shows the consequences of angina at baseline and according to the evolution of angina at 1 year in various patient subsets. Results in patients with previous myocardial infarction and multivessel disease were consistent with those of the overall population. In contrast, among patients without previous myocardial infarction, neither angina at baseline nor the occurrence of angina at 1 year was associated with higher rates of cardiovascular death or myocardial infarction, probably a consequence of more stable disease. In patients with diabetes, angina was associated with worse outcomes at baseline but not at 1 year.

## Discussion

In this observational study, angina affected approximately one-quarter of patients with stable CAD. Among these patients, anginal symptoms resolved without coronary revascularization, either spontaneously or with changes in medications, in 39.6% during the first year, with further annual decreases. Relative to patients without angina, persistence and occurrence of angina at 1 year were each associated with an increased risk of cardiovascular death or myocardial infarction, whereas resolution of angina was not. Specifically, the rate of all-cause death was higher in patients in whom angina appeared at 1 year. Lastly, fewer than 5% of patients in whom angina symptoms resolved had undergone revascularization in the prior year.

Among patients with anginal symptoms, the majority will improve over time without changes in antianginal therapy and without the need for coronary revascularization. Regression of symptoms over time in stable angina is a well-documented observation from controlled trials performed in patients with anginal symptoms^6,8,13,20,27,28^ and may reflect epicardial or microvascular adaptations to coronary disease, including development of collaterals. In the COURAGE trial (Clinical Outcomes Utilizing Revascularization and Aggressive Drug Evaluation), 58% and 72% of patients in the medical treatment arm were angina-free at 1 and 5 years compared with 13% at baseline.^27^ A similar improvement in angina was reported in the SIGNIFY trial (Study Assessing the Morbidity-Mortality Benefits of the If Inhibitor Ivabradine in Patients With Coronary Artery Disease) and the ISCHEMIA trial (International Study of Comparative Health Effectiveness With Medical and Invasive Approaches).^6,8,20^ Spontaneous regression of angina was reported before coronary revascularization and most current medical therapies became available,^29^ at a similar rate to the current study. In the present study, the largest improvement in angina was seen in the first year after enrollment, which may reflect selection bias (ie, patients with relatively recent angina may have been more likely to seek care and be enrolled) and possibly late benefits of a previous revascularization done before enrollment. Patients in CLARIFY were well-treated, with high rates of use of evidence-based secondary prevention drugs and antianginal drugs; therefore, the low rate of medical control of angina has to be interpreted in this context.^3^

Angina is a dynamic condition, and our report analyses it as such. It shows that angina evolution affects cardiovascular outcomes. Among patients without angina but with established CAD, <5% developed new angina symptoms every year. This is a high-risk group in whom intensive management may be warranted, although it is uncertain whether revascularization will result in improved clinical outcomes beyond symptom control.

Patients in whom anginal symptoms resolved had a similar rate of 5-year death to patients without angina, despite the poor prognosis associated with angina at baseline.^2,3,18,19^ Regression of symptoms could be explained by a less progressive atheromatous disease, stabilized by the use of secondary prevention drugs such as statins and angiotensin-converting enzyme inhibitors, allowing an eventless collateral development or adaptation. However, they still experienced higher rates of subsequent myocardial infarction and elective coronary revascularization, which may be related to relapse or to more invasive management despite the regression of symptoms.

Conversely, persistence of angina was associated with higher rates of cardiovascular events. This group may be heterogeneous and encompass patients with different clinical scenarios, such as older patients with severe comorbidities judged to be unamenable to revascularization, patients with refractory angina despite optimized therapy, patients with microvascular angina, or in some geographic areas, patients with typical symptoms but in whom access to coronary revascularization may be limited. At 5-year follow-up, 33.9% of patients with angina at baseline still had anginal symptoms, reflecting the unmet need for new more effective antianginal therapy.

The consequences of evolution of angina at 1 year were consistent when focusing on patients with previous myocardial infarction or multivessel disease. Among patients with diabetes, those with angina at baseline were at higher risk. This appears at odds with reports from the BARI 2D (Bypass Angioplasty Revascularization Investigation in Type 2 Diabetes) trial, in which angina status did not influence the rate of cardiovascular events and death in patients with diabetes.^30^ However, 80% of patients in the BARI 2D trial had angina or equivalent symptoms, and all had significant myocardial ischemia, a much higher rate than in CLARIFY.

It is important to note that cardiovascular adverse events were rare in the CLARIFY registry compared with previous cohorts of patients with stable CAD.^1,31^ This illustrates progress in the management of CAD in the past decade and the relatively low-risk population included in CLARIFY (which excluded patients with advanced heart failure). However, approximately 7% of patients with persisting or occurring angina at 1 year experienced cardiovascular death or myocardial infarction at 5 years; hence, these patients represent a high-risk population where active management is warranted.

Changes in angina status may be a marker for the progression or stabilization of CAD. Its occurrence or persistence might be the consequence of progression of atheromatous disease, whereas the resolution of symptoms could reflect control of CAD, achieved with medical and lifestyle management. In the ISCHEMIA trial, revascularization of ischemia-producing coronary obstructive lesions did not significantly improve cardiovascular outcomes despite improved symptomatic and functional status.^6,24^ Regression of anginal symptoms without revascularization may reflect a change in disease, whereas revascularization treats a single focal epicardial stenosis but does not affect the overall burden of CAD. This reflects the heterogenicity of angina mechanisms in patients with stable CAD, ranging from hemodynamically significant epicardial stenoses to microvascular dysfunction.^32^ The ISCHEMIA trial found that the principal benefit of routine invasive management is related to improvement in symptoms. If most patients with stable CAD have no angina, and most patients with angina are likely to experience resolution of symptoms, either with medical management or spontaneously, this further emphasizes the value of conservative management of stable CAD. However, a recent ISCHEMIA analysis showed that initial invasive management was associated with a reduction in type 1 myocardial infarction, which was itself associated with subsequent cardiovascular death.^33^

The frequency of resolution of angina in the first year suggests that trials testing antianginal drugs may need to incorporate a longer run-in period than the short 1- to 4-week duration frequently used. Furthermore, it shows that medical treatment and disease-modifying interventions may take some time to be effective and relieve symptoms, and the relatively low event rate of patients with stable CAD allows a period of watchful waiting before failure of medical management is declared. Last, our observations also emphasize the importance of sham procedures when interventions are tested to relieve angina.

### Limitations

This analysis has important limitations. Outcomes were not adjudicated and were based on investigator-reported events with dedicated questionnaires. No details on the duration of symptoms were available in yearly reports. Therefore, persistence of symptoms could reflect resolution and early relapse. Resolution of angina may reflect limitation of physical activity in some patients as shown in Table 3. However, the association of angina resolution with improvement in outcomes suggests that the former is not entirely explained by self-restriction of the patients. Angina status was missing in 5.3% of patients at 1-year and 27.3% at 5-year follow-up. However, results were consistent regardless of whether missing data were imputed. Although CLARIFY recruited in 45 countries, no patients were enrolled from the United States. Because patients were enrolled at any time after the diagnosis of CAD, there is potential inception bias and immortal time bias when studying the outcomes of patients with angina at baseline, because events occurring in patients with angina before enrollment in CLARIFY were not collected. This may have resulted in underestimation of the actual risks associated with long-standing angina. It is increasingly recognized that many patients with angina may not have obstructive CAD.^34^ Given the CLARIFY inclusion criteria, our findings do not apply to patients with angina without obstructive CAD. Further studies are needed to evaluate the coronary and microvascular changes related to angina and its evolution in patients with stable CAD.

### Conclusions

Angina still affects almost one-quarter of patients with stable CAD. In a well-treated population, anginal symptoms resolve in the majority of patients over time, most often without revascularization or changes in antianginal therapy. Changes in anginal status without coronary revascularization are associated with cardiovascular outcomes. Whereas resolution of angina is associated with improved outcomes, appearance or persistence of angina is associated with poor outcomes. Given that the benefit of antianginal medications and revascularization is restricted to symptom improvement, these observations suggest that conservative management is an effective strategy for patients with stable angina.

## Supplementary Material


