# Supplementary material for: International Observational Analysis of Evolution and Outcomes of Chronic Stable Angina: The Multinational CLARIFY Study
Source: Circulation. 2021 Jul 15;144(7):512–23. doi: 10.1161/CIRCULATIONAHA.121.054567 (PMC8360663; doi:10.1161/CIRCULATIONAHA.121.054567)
Supplement: Supplementary file 1 [file cir-144-512-s001.pdf]

SUPPLEMENTAL MATERIAL

Table of Contents

CLARIFY Registry Investigators 2
Table I. Countries in the Six Defined Geographic Areas 8
Table II. Prevalence of Angina at Baseline According to Each of the Inclusion Criteria (Not Mutually Exclusive) 9
Table III. 5-year Outcomes According to Anginal Status at Baseline 10
Table IV. Baseline Characteristics of Patients Alive With Missing Angina Status at 1 and 5 Years of Follow-Up 11
Table V. Severity of Angina at Baseline, 1-Year and 5-Year Follow-Up, in 7212 Patients with Angina at Baseline 14
Figure I. Graphic Representation of the Number of Patients Fulfilling Each of the Four Inclusion Criteria (Not Mutually Exclusive) 15
Figure II. Evolution of Angina in Patients With Stable Coronary Artery Disease Without Imputation of Missing Values 16
Figure III. Evolution of Angina in Patients with Stable Coronary Artery Disease: Full Population Scale 17
Figure IV. Sensitivity Analysis: Exclusion of Patients From Eastern Europe; Evolution of Angina in Patients With Stable Coronary Artery Disease 18
Figure V. Sensitivity Analysis: Exclusion of Patients From Eastern Europe; Clinical Outcomes According to the Evolution of Angina at 1 Year 19
Figure VI. Consequences of Angina and Evolution of Angina at 1 Year (Primary Outcome of Cardiovascular Death or Myocardial Infarction) in Subgroups 20

CLARIFY Registry Investigators

CLARIFY Executive Committee
P G Steg (chair), Paris, France; R Ferrari, Ferrara, Italy; I Ford, Glasgow, UK; KM Fox, London, UK; J C Tardif, Montreal, Canada; M Tendera, Katowice, Poland.

CLARIFY Steering Committee
Argentina: Prof. Dr. Fernando José Sokn; **Australia:** Prof. Christopher Reid; **Austria:** Prof.Irene Lang; **Belgium**: Dr. Frank Van den Branden; **Brazil:** Prof. Luis Machado César; Prof. Marco Antonio Mattos; **Brunei**: Dr. Hj. Nazar Luqman; **Bulgaria**: Prof. Assen Goudev; **Canada**: Dr Paul Dorian; **China**: Prof. Dayi Hu; **Czech Republic**: Prof. Petr Widimsky; **Denmark**: Dr. Christian Hassager; **France**: Prof. Nicolas Danchin; **Germany**: Prof. Dr. med. Stefan Kääb; **Greece**: Prof. Panos Vardas; **Gulf Countries**: Dr. Kadhim J. Sulaiman (**Oman**), Dr. Wael Al Mahmeed (**UAE**), Dr. Jassim Al Suwaidi (**Qatar**), Dr. Ibrahim Al Rashdan (**Kuwait**), Dr. Fuad Abdulkader (**Bahrain**); **Hungary**: Prof. Béla Merkely; **India**: Prof. Upendra Kaul; **Ireland**: Prof. Kieran Daly; **Italy**: Prof. Luigi Tavazzi, Prof. Roberto Ferrari; **Korea**: Prof. Yangsoo Jang; **Latvia**: Prof. Andrejs Erglis; **Lithuania**: Prof. Aleksandras Laucevičius; **Malaysia**: Dr. Ahmad Nizar Jamaluddin; **Mexico**: Prof. Marco Alcocer Gamba; **Netherlands**: Dr. Igor I. Tulevski; **Poland**: Prof. Janina Stępińska; **Portugal**: Prof. João Morais; **Romania**: Prof. Dr. Cezar Macarie; **Russia**: Prof Rafael Oganov, Prof Svetlana Shalnova; **Saudi Arabia**: Prof Muayed Al-Zaibag; **Singapore**: Dr. Mak Koon Hou; **Slovakia:** Assoc. Prof. Gabriel Kamensky; **Slovenia**: Prof. Zlatko Fras, Dr. Vojko Kanič; **South Africa**: Prof. Datshana Prakesh Naidoo; **Spain**: Prof. José Luis Zamorano; **Switzerland**: Prof. Hans Rickli, Dr. Andres Jaussi; **Thailand**: Assoc. Prof. Charn Sriratanasathavorn; **UK**: Dr. Paul Kalra; **Ukraine**: Prof. Mykhailo Lutai, Prof. Oleksandr Parkhomenko; **Vietnam**: Prof Lan Viet Nguyen; **West Indies**: Dr. Ronald Henry.

CLARIFY Investigators
Argentina: A. Ahuad Guerrero,M. Basara, F. Belcastro, J.A. Bertarini, C. Cazenave, H. Dreycopp, J. Egido, J. Estrella, D. Garofalo, J. Giordano, H. Lagioia, N. Lago, R. La Greca, L. Lema, N. Lopez Cabanillas, H. Luquez, C. Miller, E. Prada, P. Rodenas, R.G. Schena, G. Suarez, A. Tomatti; **Australia**: D.M. Colquhoun, A. Conradie, S. Cox, D. Cross, R. Fathi, B. Fitzgerald, I. Hamilton-Craig, G. Holt, S. R. Jayasinghe, N. Mai, J. Moolman, R. A. Motyer, K. Phillips, A. Rafter, A. Rahman, A. Rainbird, G. Scalia, A. Taylor, P. West, K. Alford, R. Amor, P. Astridge, B. Bastian, F. Bates, M. M. Doohan, J. Du Plooy, J. C. Ford, L. Kanagaratnam, V. Khoury, R. Parkin, J. Rogers, G. Sceats, A. Waldman, D. Wang, S. Wright, J. Ardill, P. Aylward, J. F. Beltrame, J. Bradley, W. Heddle, M. Joseph, S. Rajendran, S. Varughese, E. Brice, B. Hockings, J. Janssen, A. Kozlowski, J. O'Shea, J. Tan, D. A. Playford, K. Woollard, A. Ajani, G. Barron, N. Better, T. Carruthers, B. Chan, R. Chan, J. Cotroneo, J. T. Counsell, D. S. Eccleston, B. H. R. Forge, A. Hamer, M. Horrigan, V. M. J. Jelinek, R. Lew, D. O'Donnell, F. Panetta, M. Sebastian, P. Shetty, A. Soward, P. Srivastava, N. F. Strathmore, S. Sylivris, G. Szto, V. Veth, T. Yip; **Austria:** R. Badr-Eslam, L. Kleemann, G.Steurer, B. Mörz-Proszowski, F. Auhser, U. Teleky, G. Sepp, A. Beinhauer, D. Kerö, C. Lavicka, T. Perger, V. Hadjiivanov, M. Feldner-Busztin, R. Mika, W. Filip, A. Mahr, J. Toplak, M. G. Millauer, P. Haralambus, K. Walcher, K.H. Karner, E. Ziak, P. Painsipp, U. Frank, A. Suntinger, W. Gritsch, G. Bode, R. Herrmann, R. Raffelsberger, H. Topf, E. Moser, J. Föchterle, T. Honsig, K. Mayr, H. Mayr, R. Kaserbacher, A. Dzien, E. Galehr, M. Felbermayer, R. Schwarz; **Belgium**: R. Amini, H. Appeltants, A. Ballet, C. De Niel, G. Berkenboom, X. Bernard, T. Bouvy, M. Claeys, Y. Dascotte, L. Davin, T. De Backer, A. De Meester, S. De Ridder, P. Dendale, K. Denef, M. Emonts, J. T. M. Geraedts, M. Goethals, J-M. Grégoire, T. Herbots, E. Hoffer, W. H. J. Hutse, P. Lafontaine, P. Lefebvre, H. Lesseliers, G. Odent, A. Pasquet, B. Peperstraete, P. Purnode, A. Rogowsky, M. Rosseel, J-P. Salembier, P. Surmont, A. M. F. Vandeplas, S. Van de Walle, F. Van den Branden, P. Vandergoten, B. G. Vanhauwaert, L. Vanneste, J. Vercammen, D. Verleyen, D. Vermander, C. Weytjens; Brazil: A. Rocha de Lorenzo, A. Felice Castro Issa, B. Mahler Mioto, C. de Brito Vianna, C. Okawabata, E. Giusti Rossi, F. Fernandes, F. Pitella, F. Henpin Yue Cesena, J. F. Monteiro Ferreira, J. F. Junior, L. Tonet, L. Machado Cesar, L. H. Gowdak, M. A. Matos, M. Moretti, R. Vicente Amato, R. Tadeu Munhoz, S. R. Coimbra; **Brazil:** A. Rocha de Lorenzo, A. Felice Castro Issa, B. Mahler Mioto, C. de Brito Vianna, C. Okawabata, E. Giusti Rossi, F. Fernandes, F. Pitella, F. Henpin Yue Cesena, J. F. Monteiro Ferreira, J. F. Junior, L. Tonet, L. Machado Cesar, L. H. Gowdak, M. A. Matos, M. Moretti, R. Vicente Amato, R. Tadeu Munhoz, S. R. Coimbra; **Brunei**: H. N. Luqman; **Bulgaria**: S. Yakovova, M. Mantcheva, V. Mincheva, L. Baurenski, K. Karastanev, V. Yordanova, Y. Peneva; **Canada**: P. Wong, M. Fagan, G. Sabe-Affaki, F. M. Villasenor, W. K. Son, D. E. Manyari, N. Giacomantonio, B. J. Lubelsky, D. Ezekiel, J. C.S. Leong, A. Grover, J. Vavougios, Y. Pesant, A. M. Kushner, M. M. W. Yeung, G. E. Vertes, F. J. Nasser-Sharif, D. Spensieri, A. Roy, T. T. Nguyen, M. Leclair, P. Morra, C. Everton Biglow, J. F. Baril, K. Lai, D. S. Wong, V. Martinho, G. A. Antoniadis, G. R. Searles, D. Rouse, G. Brisson, S. King Wong, R. S. Collette, M. S. C. Ho, C. Constance, R. Gendreau, G. W. Kellam, T. A. Cieza Lara, H. A. Boyrazian, M. Shamsuzzaman, D. R. Spink Jr., A. P. T.Wong, J. Janes, M. Czarnecka, D. Saulnier, G. Levesque, P. F. Clavette, A. Kokis, T. L. Orenstein-Lyall, A. Shekhar Pandey, J. Robb, G. Verret, W. Czarnecki, F. Perreault, G. Chouinard, G. Lafrance, G. M. Fullerton, P. LeBouthillier, Q. H. Tran, I. Rodriguez Marrero, F. B. Ramadan, P. Talbot, M. A. Fazil, J. Yi-Ming

Cha, S. Garg, R. Chehayeb, B. Roy, Y. K. Chan, H. E. Harlos, H. B. Matheson, R. Patel, G. F. Vaz, J. S. Bhatt, E. Liu, T. H. Ashton, H. Sullivan, L. P. Quinn, K. Yared, A. K. Gupta, B. Sullivan, J. Campbell, S. Pallie, H. H. Kim, D. Savard, J. M. Cherry, J. Gold, G. Brouillette, A. Belanger, J. Berlingieri, W. Nisker, G. Boutros, A. I. Bakbak, L. Lasalle; **China**: LL. Chen, MH. Chen, MY. Chen, XP. Chen, YD. Chen, HL. Cong, SF. Ding, JT. Dong, SH. Dong, ZM. Du, LL. Feng, W. Gao, H. Ge, SJ. Ge, T. Guo, Y. Guo, Z. Huang, Y. Huo, HG. Jin, YN. Ke, HW. Li, HM. Li, ZC. Li, YJ. Li, JL. Liu, QL. Liu, SW. Liu, SZ. Lv, W. Miao, GZ. Pan, XD. Pu, ZM. Qian, X. Su, JH. Tao, HP. Wang, JA. Wang, NF. Wang, T. Wang, W. Wang, Y. Wang, M. Wei, SY. Wu, YJ. Wu, LG. Xiong, D. Xu, J. Yan, K. Yang, M. Yang, TL. Yang, XL. Yang, JM. Yu, HS. Zeng, H. Zhang, HQ. Zhang, L. Zhang, SX. Zhou, YL. Zhou. **Czech Republic**: J. Bozkova, J. Carda, S. Dedkova, A. Dufka, J. Fridrich, T. Hodac, R. Jirmar, A. Kadleckova, M. Karlicek, J. Krupicka, J. Kuchar, V. Lavicka, J. Leso, Z. Lorenc, M. Micko, P. Navratil, I. Petrova, P. Povolna, L. Raisova, P. Raska, V. Ravlyk, S. Schlesingerova, E. Smrckova, P. Sternthal, H. Stursova, P. Vymetal, **Denmark**: P. Wiggers, J. Markenvard, L. K. Andersen, L. Frost, J. Refsgaard, S. Strange, K. Egstrup, R. Sykulski, P. Hildebrant, T. Haghfelt, M. Ege, L. Saaby; **France**: S. Cattan, M. Adam-Blanpain, M. Adda, N. Aimouch, L. Ardouin, S. Assouline, A.Aumjaud, C. Barjhoux, R. Baroudi, C. Beaurain, M. A. Bennouna, A. Bernard, C. Bernardeau, E. Blanc, I. Blum-Decary, G. Bodur, C. Boesch, J. Bonal, R. Bonhomme, J. L. Bonnet, J. Bories, M. L. Bourachot, F. Brumelot, M. Brunehaut Petaut, C. Brunschwig, P. Buffet, P. Calmettes, I. Centa, B. Chartier, P. Chemin, F. Chometon, J. Cohen, R. Colin, Y. Cottin, F. Crespo, A. Dabboura, F. David, P. Dehayes, P. Dematteo, O. Dibon, P. Dodemant, V. Dormagen, X. Dreyfus, J. M. Dubois, F, Duclos, M. Ducoudre, O. Duprez, P. Durand, E. Durand, P. Egloff, M. Escande, M. C. Escourrou Berdou, G. Esna Ashari, I Feldmann, J. Ferrieres, E. Foltzer, B. Fontanet, M. Garandeau, T. Garban, S. Geffroy, T. Gillet, S. Godart, P. Gosse, P. Gratia, O. Greiner, A. Gueusquin, E. Guiu, J. M. Guy, S. Haddad, V. Hennebelle, S. Honorat, A. Hourany, G. Hua, P. Jacquier, S. Jean, R. Jeremiasz, P. Kohler, A. Lacroix, M. Leandri, Y. Lemiere, M. Liautard, P Loheac, J. C. Louchart, P Magnus, B, Maheu, H. R. Malaterre, G. Manchet, J. Mantoux, D.Manzi, M. Marachli, M. Maroun, N. Meneveau, E. Messas, J. L. Mougeolle, T. Mouhat, J, J. Muller, M. Naisseh, P. Nocon, D. Onger, A. Ouguoujil, M. Ovize, E. Page, K. Pareathumby, A. Pleskof, P. Poinson, G. Pons, P. Pouderou, J. N. Poujois, V. Probst, F. Prunier, L. Prunier, V. Puel, D. Rechtman, R. Rennert, B. Rijavec, Y. Riou, J. Robert, C. Roche, G. Roul, B. Salaun, B. Saleh, A. Sandalian, M. Sander, A. Schenowitz, A. Silvestre, H. Soleille, S. Tabet, M. Tardy, F. Thomas-Richard, B. Truong, J. Varaldi, H Vial, J. M. Walch, M. Wazana, R. Zeitouni, H. Audibert, F. Alizon, A. Amlaiky, M. Asplanato, C. Baranes, M. Bariaud, F. Bernasconi, P. Bousquet, C. Ceraulo, G. De Geeter, J. Donetti, B. Doucet, J. Doucet, T. Dutoya, D. Ennouchi, M. H. Fallacher, G. Fouquet, V. Fourchard, J. Gdalia, G. Grollier, S. Guerard, P. A. Jeannerat, Y. Jobic, V. Joulie, P. Jourdain, V. Jouve, R. Ketelers, G. Khaznadar, P. Kohan, B. Koujan, B. Lammens, I. Landragin, E. Le Moal, D. M'Bey, F. Maes, S. Maheas Morlet, R. Massabie, D. Meddah, F. X. Meriaux, C. Mestre-Fernandes, P. Meyssonnier, M. Migliore, J. Milewski, J. F. Millet, S. Mingam, P. Nazeyrollas, F. Paganelli, F. Pellerin, F. Petitjean, A. Pinzani, A. Pladys, P. Primot, A. Pucheu, A. Rahali, P. Ravoala, D. Rousson, P. Samama, M. Sardon, R. Silvestri, P. Soskin, X. Tabone, C. Tricot, B. Vaquette, M. Vogel, M. Weingrod, V. Aboyans, R. Amoretti, J. Aubry, P. Berthezene, D. Binet, X. Bonnaud, P. Bonnet, A. Bonny, T. Bouchaya, C. Boureux, J. M. Bourgeois, L. Brottier, B. Cavert, S. Cleron, E. Dechoux, C. Delhomme, J. P. Detienne, J. P. Dubs, B. Faudon, F. Fellous, R. Fressonnet, Y. Garaud, D. Garcia, M. Geneves, J. L. Gleizes, C. Guyetand, B. Hermellin, D. Iovescu, J. P. Kanner, P. Khanoyan, A. Leherissier, A. Maximovitch, B. Merian, P. Messali, Y. Moreau, J. Moyal, L. Payot, L. Petoin Peuch, J. L. Prevot, P. Raymond,D. Relange, S.Reymond, J. F. Robert, H. Rosenstein, J. Schneider, R. Schultz, P. Tanielian, F. Thoin, L. Thomas, P. Touzet, G. Steg, G. Amiel Oster Sauvinet, F. Baylac Domengetroy, K. Chamou, B. Etcheverry, J.L. Farges, J. Y. Fraboulet, M. Goralski, D. Janody, B. Mamez, W. Manlay, F. Paillard, F. Pelier, A. Petit, M. Skonieczny, R. Augarde, J. B. Fournier, S. Liandrat, P. Lim, A. I. Noury, D. Paris, M. Saade, J. M. Stordeur, N. Danchin, M. Pornin, L. Fauchier, M. Galinier, M. A. Balice-Pasquinelli, P. Sosner, S. Yvorra, E. Delcoulx, F. Mouquet, J. E. Poulard, A. Sudre, P. Heno, F. Biausque, M. Guenoun, G. Attia, S. Pouwels, L. Carpentier, E. Verbrugge, C. Ziccarelli, M. Elkohen, J. Tricoire, P. Lang, O. Huttin; **Germany**: B-M. Altevogt, U. Altmann, M. Baar, S. Berrisch-Rahmel, A. Birkenhagen, I. Bläse, R. Blindt, R. Bosch, A. Brattström, H-H. Breuer, M. Castrucci, S. Cicek-Hartvig, R. Cierpka, M. Claus, M. Deissner, M. Drexler, T. Eggeling, G. Eisele, D. Enayat, S. Frickel, S. Gessner, K. Giokoglu, J. Gmehling, F. Goss, P. Grooterhorst, D B. Gysan, R. Haberl, W. Haerer, N. Hassler jun, S. Heinemann-Meerz, F. Henschel, M. Hinrichsen, W. Hofer, A. Hofmeister, G.Hoh, E. Horstkotte, F. Jäger, M. Jeserich, U. Keil, H. Killat, S. Kimmel, M. Kindel, P. Kindler, S. Kleta, J. Könemann, K. König, H. Krause-Allmendinger, K. Kronberg, I. Kruck, V. Männl, A. Meinel, G. Mentz, E. Meyer-Michael, F. Mibach, S. Möller, S. Muth, E. Nelböck-Huber, D. Ohlmeyer, Z. Özkan-Rashed, C- P. Paulus, S. Perings, J. Placke, C. Raters, N. Reifart, A. Rink, K. Rybak, I. Salecker, K-H. Schermaul, U. Schlesinger-Irsch, E. Schmidt, K-H. Schmitz, N. Schön, T. Schröder, B. Sievers, M. Simon, U. Spengler, M. Speth-Nitschke, A. Stumpp, S. Szabo, J. Taggeselle, A. Tamm, A. Thelemann, C. Thelemann, H. Thümmel, G. Unger, A. Utech, J. Volmar, B. Wauer, G. Wehr, L. Weinrich, R.Weinrich, U. Windstetter, J H. Wirtz, N. Wittlich, P. Ziehn, P. Zündorf; **Greece**: C.Avgerinos, O. Gouli, D. Stergiou, I. Alexopoulos, C. Pappas, I. Petropoulos, G. Chatzioakim, N, Pontikakis, C. Priftis, P. Mpompoth, I. Bourazanis, A Papathanasioy, S. Avlonitis, C. Zakopoulos, G. Koutsimpanis, I. Tsamopoulos, C. Christoforidis, V. Zachos, P. Kalaras, M. Karachaliou, C. Liatas, G. Pournaras, G. Theodorakis, I. Orestis, K. Panisois, E.

Chalkiadakis, V. Arfaras, G. Kolios, P. Boutsikos, A. Kotsalos, D. Mitropoulos, A. Samothrakitis, K. Svolis, E. Anastasiou, T. Gkinis, P. Dalampyras, A. Kalampalikis, I. Leontaridis, S. Gabriilidis, I. Konstantinidis, V. Plastiras, P. Tarenidis; **Gulf Countries**: **Oman**: Y. Al Wahshi, P. P. Singh, A. Narayan, F. Al Tamimi, J. Al Yazeedi, M. Ayche, A. Al Lawati, M. Al Dhanki, **United Arab Emirates:** A. Salustri, T. Salah, M. Y. Tamimi, A. Agrawal, A. Wassef, F. Baslaib, G. Al Radaideh, A. Yusufali, N. Bazargani, **Kuwait:** M. Akbar, H. Abdel Wahab, S. Abdel Malak, I. Ghaly, H. Hafez, F. Al Kandari, M. Haiba, M. Alanbaei, **Qatar:** M. M. Gomaa, **Bahrain**—A. Khalifa; **Hungary**: I. Marozsán, I. Édes, I. Czuriga, A. Cziráki, K. Tóth, Á. Dongó, P. Túri, T. Forster, J. Borbola, B. Bachmann, G. Masszi, M. Orbán, G. Gerges, G. Balogh, É. Bajcsi, I. Takács, L. Nagy, B. Kisjós, A. Jánosi, A. Nagy, K. Nagy, A. Büttl, J. Lippai, Zs. Sziegl, Zs. Malkócs, A. Földi, K. Fikker, E. Szabó, R. Forrai, Z. Sebõk, Prof. B. Merkely; **India**: R. Gupta, S. Natarajan, J. Dalal, R. K. Saran, A. Mehta,M. P. Samal, I. A. Khan, T. Ghose, J. P. S. Sawhney, T. Roy, S. Chandra, S. Modi, M. M. Singh, G.Vijayaraghavan, L. Sreenivasa Murthy, S. S. Ramesh, Dr Dayasagar Rao V, M. S. Chenniappan, A. Vadavi, K. Kunhali, K. Srinivasa Reddy, Su. Thillai Vallal, P. Khera, B. Prasad, D. Shukla, A. K. Trivedi, R. Ahuja, J. Rawal, R. Karnik, M.S. Hiremath, D. K. Kumbla, S. R. Shetty, N. S. Chonkar, Late MJuneja, B.K. Goyal; **Ireland**: R. Sheahan, C. Daly, C. Vaughan, S. Fleming, P. Shiels, P. Keelan, T. Kiernan, B. Day, K. Kelly, F. MacNamara, B. Maguire, A. Clifford, A. O'Gara; **Italy**: G. Guardigli, G. Pes, G. Caridi, A. Frattola, B. Doronzo, G. Riccioni, A. Lacchè, F. Massari, S. Orazi, D. Carretta, M. Provvidenza, R. Pedretti, A. Nicolino, S. Felis, V. Pernice, A. Gaglione, P. Gori, P. Martina, V. d'Alessandro, F. Giacomazzi, P. Terrosu, C.Cernetti, R. Antonicelli, G. Ansalone, M. Balbi, C. Tamburino, L. Moretti, S. Tantillo, F. Patriarchi, F. Proietti, G. Sinicropi, G. Maragoni, V. Mallamaci, D. d'Este, P. Azzolini, E. Brscic, S. Bongo, A. Gigantino, G. Perna, M. S. Mayer, C. La Rosa, G. Muscio, V. Scollo, F. Magliari, C. Petrillo Pio, M. Castellari, P. Di Pasquale, Dr Scalzo, F. Saporito, N. Capuano, F. Alitto, N. Marchionni, M. Turiel, A. Bianco, C. Greco, L. Marullo, R. Testa, A. Vicentini, S. Novo, F. La Varra, L. Tavazzi, M. R. Conte, Z. Lazerevic, F. Colivicchi, C. Macchi, J. Dalle Mule, G. Sibilio, A. Achilli, C. Proto ; **Korea**: S.M. Kang, B. K. Koo, S. K. Hong, W. Kim, S. H. Lee, B.S. Yoo, H. S. Seo, H. C. Gwon, D. H. Kang, H. M. Kwon, I. H. Chae, S. J. Oh, J. H. Shin, C. W. Goh, Y.S. Byun, J. H. Zo, T. J. Hong, D. S. Kim, T. J. Cha, J. K. Ryu, Y. J. Kim, J. Y. Hwang, S. H. Hur, M. H. Jeong, S. K. Oh, D. K. Jin, K. T. Jung, J. Y. Rhew, S. Lee, D. W. Jeon, S.J. Yoon, S. H. Kim; **Latvia**: I. Mintale, G. Latkovskis, S. Hansone, N. Rozkova, A. Baika, I. Jasinkevica, S. Abele, I. Laizane, N. Pontaga, V. Ecina, I. Mihailova, A. Kondratovica; **Lithuania**: A. Laucevičius, R. Jurgaitienė, R. Šlapikas, G. Barauskienė, E. Jankauskienė, S. Revienė, D. Zaronskienė, O. B. Šlapikienė, N. Kupstytė, E. Rinkūnienė, R. Steponėnienė, J. Kojelienė, J. Badarienė, V.Dženkevičiūtė, E. Sadauskienė, I. Butkuvienė, R. Stankevičius, R. Paliulionienė, R. Snikytė, R. Mažutavičius; **Malaysia**: A. N. Jamaluddin, A. A. Abdul Rahim, A. K. Mohamed Yusof, K. H. Chee, M. A. Sadiq, S. Ramanaidu, K. H. Sim, T. K. Ong, A. Y. Y. Fong, B. C. Chang, S. K. Chua, Y. L. Cham, N. H. Mohd. Amin, S. K. Tan, N. Z. Khiew, A. Said, C. K. Abdullah, Y.W Cheah, J. Sinnadurai, K. K. H. Lau, C. K. Choor, K. K. Sia, C. C. Ang, J. S. Awtar Singh, M. Z. Abdul Wahab, C. K. Wong, A. K. Ghapar, A. Muthu, K. A. Mahendran, A. H. Jaafar, K. H. Ng, A. I. Ruhani, H. A. R. Tahir, H. Abdul Manap, B. S. K. Ch'ng, E. T. Ch'ng, A. S. Abdullah, O. Ismail, A. S. Sahar, B. B. Abdul Kareem, M. A. Sheikh Abdul Kader, S. K. Ma, K. K. Chan, T. H. Goh, A, Singh, H. B. Liew, C. M. Chu, R. K. M. Bhaskaran, R. P. Shah, K. L. Joseph, H. Noor Hasni, W. K. Ng, G. H. Choo, N. Saaidin, C. K. Yeo, V. M. Lai, Y. C. Lai, M. H. Tay, B. A. Lim; Brunei: H. N. Luqman; **Mexico**: Guillermo Llamas Esperon, J. de Jeús Zuñiga Sedano y America Alvarez, F. Azar Manzur, C. Jerjes Sánchez, J. Cerda Rojas, J. Carrillo Calvillo, F. Petersen Aranguren, C. Martínez Sánchez, A. Álvarez Sangabriel, G. Vieyra, S. González Romero, A. Puente Barragán, F. Redding Escalante, J. Chávez Paez, E. Fernandez Valadez, E. Gaxiola, L. E. Manautou, O. Henne Otero, M. Barrera Bustillos, J. L. Leyva Pons, E. Gómez Álvarez, J. R. Romo Santana, J. Martínez Redding, A. Arias Mendoza, I. Rodríguez Briones, J. de Jesús Rivera Arellano, J. L. Arenas León, M. Alcocer Gamba, E. Alexanderson, M. E. Ruíz Esparza, L. A. Elizondo Sifuentes, J. L. Briseño, E. Sandoval Rodriguez, M. L. Fuantos Delgado, S. Sandoval Navarrete, U. Casas Juarezy, A. Loera Pinales, A. Castro, R. Cue Carpio, E. Rodríguez, G. Rojas, G. Solache, R. Díaz Juárez, R. Baleón, C. Ferreyra Solorio, H. A. Ramírez Reyes, M. López Martínez, M. A. Romero Maldonado, J. Escobedo de la Peña, J. Hilario Jiménez Orozco, F. A. Reyes Cisneros, J. Álvarez Gil, G. Bautista López, M. Odín de los Ríos Ibarra; **Netherlands**: I. I.Tulevski, G. A. Somsen, K. Miedema; **Poland**: I. Chlewicka,P. Brodzicki, T. Stasiuk, P. Szałkowski, W. Kulig, M. Maliszewski, K. Królicka, J. Zdrojewska, I. Nikodemska, A. Szpak, M. Wrębiak-Trznadel, A. Prokop, M. Szulc, A. Olszewski, W. Kępa, J. Banach, M.Węglarz, A. Gałuszka-Bilińska, A. Królak, E. Cisowska-Drozd, K. Orzechowski, M. Jeżewska, K. Adamaszek, G. Glanowska, T. Pitsch, G. Matuszewska, A. Nowowiejska-Wiewióra, M. Dereń, G. Walawski, M. Sołtysiak, R. Wysocki, G. Jarosiński, A. Drzewiecka, T. Ługowski, A. Jankowska, P. Błaszczak, J. Drozd, E. Łotocka, R. Duchowska, D.Sobczyk, P. Jarmużek, M. Sidor, D. Adamczyk-Kot, J. Sudnik, J. Cygler, I. Skoczylas, B. Poprawa, L. Kisiel, U. Kossowska, B. Sikorska-Buczkowska, K. Modzelewska, B. Demianiuk, W. Streb, T. Mularek-Kubzdela, P. Bogdański, E. Kaźmierczak, R. Zimoląg, J. Lorenc, R. Furtak, A. Regulska, M. Winter, M. Fic, P. Turek, E. Nowicka, W. Bryl, L. Lenartowska, O. Jerzykowska, M. Maćków, W. Gadziński, R. Kacorzyk, D. Zalewska, R. Sadłowski, J. Słaboszewska, M. Gruchała, A. Frankiewicz, J. Walczewska, A. Adamkiewicz-Piejko, R. Chyrek, L. Jankowska; **Portugal**: A. Correia, A. Girão, Á. Herdade, A. Sequeira, A. Tavares E Taveira, A. Gonzaga, A. Ribeiro, A. Albuquerque, A. Fernandes, A. Estriga, A. Rocha De Almeida, A. Lourenço, A. Pereira, A. Faria, B. Carvalho De

Moura, C. Camossa, C. Alves, C. Aguiar, C. Rodrigues, E. Wellenkamp, F. Fernandes De Sousa, F. Moreira Pinto, F. Matias, G. Silva Alves, G. Bragança, G. Proença, G. Mariano Pêgo, H. Vinhas, I. Arroja, J. Morais, J. Silva E Sá, J. Vasconcelos, J. Matos, J. Freitas, J. Ferreira, J. Costa, J. Alcaravela, J. Mimoso, J. Antunes, J. Ferreira Dos Santos, J. Nobre Dos santos, J. Fernandes, J. Chambel De Aguiar, J. Moreira, J. Carvalho, J. Forte De Carvalho, J. Calaça, L. Simões, L. Lopes Antunes, L. Soares, L. Semedo, L. Macedo, L. Sargento, L. Basto, L. Rebelo, L. Oliveira, M. Catarino Carvalho, M. Alves Costa, M. C. Gamboa, M. F. Ferrão E Vasconcelos, M. H. Custódio, M. I. Mendonça, M. J. Pinto Vaz, M. Espiga De Macedo, M. Lazaro, M. Martins Oliveira, N. Pelicano, N. Lousada, O. Rodrigues, P. Matos Dias, P. F. Fonseca, P. Ferreira, P. Farto E. Abreu, P. Monteiro, R. Seabra Gomes, R. Carvalho, R. Santos, R. Rosado Soares, S. Baptista, S. Reis Monteiro, V. Gil, V. Sanfins, V. Martins; **Romania**: M. Anghel, C. Arsenescu Georgescu, K. Babes, M. Banu, R. Beyer, I. Bratu, A. Bumbu, R. Capalneanu, O. D. Chioncel, T. Chiscaneanu, R. Christodorescu, N. Cindea Nica, M. Cinteza, S. Coman,M. Constantinescu, E. Craiu, G. A. Dan, D. C. Dan, A. Dan, C. M. David, M. Dorobantu, D. Farcas, V. Firastrau, C. Florescu, A. Ghicu, A. Giuca, R. Grigoriu, D. D. Ionescu, L. C. Iosipescu, M. V. Ivan, D. Lighezan, S. Magheru, M. Magherusan, S. M. Marinescu, A. C. Motoc, R. Musetescu, M. Rau, L. Rotaru H. Rus, O. Sirbu, L. Sorodoc, C.M. Spinu, G. Stanciulescu, C. Statescu, M. Toringhibel, R. Trambitas, N. Trocan, A. Tudose, D. Vinereanu, M. Zagreanu, R. Motomancea, C. Militaru; **Russia**: D. Dymova, N. Semenova, A. Zherebtsova, V. Fedoskin, N. Gurianova, N. Bolotova, V. Knyazeva, T. Spitsina, N. Sytilina, N. Atamanchuk, M. Giorgadze, S. Zarechnova, S. Kutuzova, Y. Sharapova, I. Stelmakh, O. Sinyukova, S. Rostik, L. Evtukhova, L. Sukhanova, T. Makhieva, S. Tereshko, V. Kolesnikov, E. Kochurov, B. Marchenko, S. Nurgalieva, Z. Galeeva, E. Andreicheva, V. Zakirova, L. Baleeva, A. Minsafina, N. Borodina, Y. Arkhipova, T. Krechunova, M. Scherbak, A. Merkhi, N. Aksyutina, O. Ratovskaya, E. Suglobova, Y. Kozhelenko, E. Potapova, G. Poluyanova, N. Naberezhnova, E. Daniels, K. Atueva, L. Tsaryabina, A. Kurekhyan, N. Khishova, E. Dubinina, O. Demina, P. Mochkina, E. Bukanina, S. Tolpygina, Y. Polyanskaya, A. Malysheva, T. Kheliya, A. Serazhim, V. Voronina, Y. Lukina, R. Dubinskaya, N. Dmitrieva,M. Kuzyakina, N. Khartova, N. Bokuchava, E. Smirnova, A. Esenokova, Y. Pavlova, O. Smirnova, P. Astrakhantseva, S. Bykovskaya, O. Charikova, K. Berdnik, T. Karaseva, L. Zhabina, N. Oleinikova, O. Dzhkha, S. Grigoryan, E. Yakovenko, T. Ivaschenko, I. Kiseleva, T, Shokina, M. Novikova, A. Khodanov, L. Popova, L. Latyntseva, O. Kilaberiya, K. Makarenkova, N. Nosova, T. Gerasimova, L. Boikova, N. Sharapova, Y. Kulikova, N. Pasechnaya, E. Bulakhova, S. Kurochkina, I. Bratishko, O. Likhobabina, E. Panova, N. Voronina, N. Bizyaeva, O. Gusev, N. Nevolina, T. Arsentieva, I. Budanova, E. London, Melnikova, A. Khripun, L. Polyaeva, E. Osadchuk, O. Krasnoslobodskaya, N. Yakimova, A. Lugin, Y. Sosnova, E. Il'ina, G. Kositsina, I. Shanina, S. Kostomarova, M. Malgina, M. Omelchenko, I. Gorlova, S. Eidelman, A. Salakhova, B. Bondarenko, R. Sopia, N. Baboshina, N. Eliseeva, F. Tumarov, N. Petrochenko, I. Khudina, N. Arabadzhi, V. Samakhovets, L. Tkhorzhevskaya, T. Sinotova, E. Zherlitsyna, S. Minkin, N. Petrova, Y. Tikhonov, N. Shmakova, V. Abduvalieva,M. Kuzmicheva, L. Nikolaeva, O. Varezhnikova, T. Dmitrieva, E. Mikhailova, Y. Yanina, L. Kapustina, Z. Vazhdaeva, G. Golovina, N. Fedorova, I. Nikolaeva, O. Fillipova, L. Gareeva, F. Tuktarova, N. Khmelevskikh, V. Karnot, M. Golub, I. Surovtseva, V. Kulygina, N. Shelomova, I. Kruglova, I. Pokrovskaya, O. Rodina, L. Polkina, N. Biryukova, E. Filippova, E. Kotova, T. Ignatieva, T. Alekseeva, L. Gruznykh, E. Mozerova, E. Moksyuta, E. Kosachek, N. Srtumilenko, O. Baranova, T. Voronova, L. Bayakhchan, I. Grudtsina, L. Gorshkova, O. Shamsutdinova, M. Getman, I. Gorodilova, N. Karnaukhova, V. Rotenberger, L. Isaeva, G. Lebischak, V. Ryzhkova, E. Usoltseva, D. Mescharekova, E. Tavlueva, E. Mineeva, M. Stikhurova, L. Kosareva, O. Grechishkina, S. Nikishina, A. Ilyukhina, O. Gureeva, I. Soin, S. Erofeev, S. Lebedev, l. Kudryavtsev, E. Gamzatov, N. Maximchuk, L. Grekhova, L. Kolevatova, M. Kazakovtseva, O. Kolesova, L. Zharikova, V. Kukaleva, N. Starostina, I. Grushetskaya, V. Kazachkova, I. Pashentseva, S. Shimonenko, I. Sirazov, A. Chernozemova, O. Golubeva, S. Mingalaeva, E. Zatsarina, D. Kozlov, N. Davydova, O. Larina; **Saudi Arabia**: K.F. Alhabib, A. Hersi, H. Al-Backer, H. AlFaleh, A. Mobeirek, M. Arafah, M. Al-Shamiri, F. El-Shaer, M. Al Zaibag, M. Bdeir, I. Suliman, A. Mukhtar, H. Omar, A. Jamiel, A. Elkrail, M. Alanazy, M. Habab, K. Ashmak, R. Nourallah; **Singapore**: K. H. Mak, B. Singh, T. S. Chee, C. C. Koo, L. P. Low, V. P. Nair, K. S. Ng, S. S. S. Quek, E. H. M. Tan, A. L. R. Ng, H. H. Chuang; **Slovakia**: G. Kamensky, G. Kaliska, J. Murin, K. Hatalova, L. Gaspar, I. Simkova, J. Dubrava, J. Pjontek, D. Pella, A. Banikova, M. Szentivanyi, F. Kovar, J. Benacka, I. Gonos, F. Fazekas, P. Kycina; **Slovenia**: J. Poles, Z. Fras, A. Pernat, A. Veternik, N. Černič-Šuligoj, M. Kerbev, I. Krajnc, P. Zagožen; **South Africa**: A. Alam, B Brown, B. Luke, E. Variava, R. Nethononda, S. Joubert, P. Matthews, L. Nkombua, V. Antia, D. P. Naidoo, J. Bhayat, S. K. George, N. Ranjith, G. H. M. Vawda, S. Govender, I. Soosiwala, K. Shein, M. Panajatovic, J. Flores, M. S. H. Khan, S. Blignaut, K. Coetzee, L. Burgess, V. Freeman, H. D. Theron; **Spain**: M. A. Arnau, Vives, F. J. Abardía Oliva, P. Aguar Carrascosa, V. Albero Martínez, J. M. Alegret Colomer, E. Alegría Ezquerra, C. A. Almeida Fernández, N. Alvarenga Recalde, A. Alvarez Auñon, P. Alvarez García, C. Amo Fernández, C. Amoros Galito, R. Ancín Viguiristi, M. Aparici Feal, A.Ardiaca Capell, J. Arnedillo Pardo, G. Arquero García, V. Arrarte Esteban, M. Baquero Alonso, P. Barahona Pérez, J. L. Bardají Mayor, V. Barriales Alvarez, A. Batalla Celorio, B. Berzal Martín, D.Bierge Valero, J. Blanco Castiñeiras, F. Bosa Ojeda, C. Botana Penas, H. Brufau Redondo, J. Bruguera Cortada, R. Cabrera Solé, F. Calvo Iglesias, S. Cantabrana Miguel, R. Carrillo Cardoso, M. Casanovas Pié, P. Casas Giménez, E. Castillo Lueña, J. A. Castillo Moreno, M. Castillo Orive, A. Chirivella González, J. M. Chopo Alcubilla, V. Climent Payá, M. A. Cobos Gil, J. L. Colomer

Martín, A. Concepción Clemente, R. Cortés Sánchez, D. Cremer Luengo, S. Darnes Soler, J. de Andrés Novales, R. De Castro Aritmendiz, J.de Juan Baguda, M. de los Reyes López, J. L. Delgado Prieto, J. L. Díaz Díaz, C. Escobar Cervantes, J. Ezcurdia Sasieta, L. Facila Rubio, C. Falces Salvador, P. Federico Zaragoza, R. Fernández Alvarez, F. Fernández de la Cigoña, L. A. Fernández Lázaro, L. C. Fernández Léoz, R. Fernández Mouzo, M. Fernández-Valls Gómez, B. Ferreiro Rodríguez, C. Franco Aranda, J. Freire Corzo,J. Fuertes Alonso, J. Fuertes Beneitez, E. Galve Basilio, C. García García, M. J. García Martínez, MJ. García González, S. García Ortego, C. García Pindado, V. García Saavedra, J. García-Moll Marimón, R. Gascueña Rubia, D. Gentille Lorente, H. Gervas Pavón, R. Gilabert Gómez, J. J. Gómez Barrado, J. J. Gómez Doblas, M. J. Gómez Martinez, C. González Juanatey, V. González Toda, M. Gonzalvez Ortega, E. Gordillo Higuero, J. Hernández Afonso, D. Herrera Fernández, E. Homs Espinach, A. Idoate Gastearena, M. Irurita Latasa, R. Izquierdo González, M. Jaquet Herter, M. Lagares Carballo, J. A. Lastra Galán, B. Limeres González, M. A. López Aranda, L. López Barreiro, D. López Gómez, A. López Granados, V. López Mouriño, J. L. López-Sendón, M. Luaces Méndez, L. Mainar Latorre, E. Marín Araez, F. Marín Ortuño, A. Martín Santana, J. Martínez Florez, J. Martínez González,J. F. Martínez Rivero, D. Marzal Martín, G. F. Mazzanti Mignaqui, A. Melero Pita, E. Molina Laborda, G. Miñana Escrivá, V. Montagud Saavedra, Mª A. Montero Gaspar, J. Mora Robles, J. Morales González, J. Moreno Arribas, Mª T. Moreno Casquete, C. Moya López, N. Murga Eizagaechevarría, F. Narro García, J. Navarro Manchón, C. Navas Navas, E. Novo García, J. A. Núñez Gamero, A. Ordóñez España, J. A. Ortiz de Murua López, E. Orts Soler, E. Otero Chulian, L. Pastor Torres, A. J. Paule Sánchez, M. A. Paz Bermejo, V. Pedrosa del Moral, G. Peña Pérez, J. Á. Perea Egido, L. Pérez de Isla, S. Pérez Ibiricu, Mª A. Pérez Martínez, M. Pérez Paredes, E. Peris Domingo, A. M. Peset Cubero, J. Pinar Sopena, C. Pindado Rodríguez, Mª J. Pinilla Lozano, C. Piñero Ramírez, Y. Porras Ramos, E. Prieto Moriche, F. Ramos Ariznabarreta, M. Rayo Gutiérrez, J. M. Roca Catalán, A. Rodríguez Almodóvar, J. Rodríguez Collado, A. Rodríguez Fernández, J. A. Rodríguez Fernández, J. A. Rodríguez Hernández, I. Rodríguez Tejero, I. Romeo Castillejo, D. Romero Alvira, J. A. Romero Hinojosa, C. Romero Menor, P. Rossi Sevillano, E. C. Rueda Calle, J. Rueda Soriano, P. Ruiz Pérez, T. Sagastagoitia Gorostiza, I. Sainz Hidalgo, M. Sandin Rollán, S. Santaolalla Rodríguez, E. Santas Olmeda, J. L. Santos Iglesias,M. L. Sanz Rodríguez, I. Segura Laborda, S. Serrano García, B. Sevilla Toral, L. Silva Melchor, E. Simarro Martín-Ambrioso, R. Sola Casado, C. Soriano Navarro, Mª I. Soto Ruiz, P. Talavera Calle, E. Teijeira Fernández, P L. Torres Díaz, A. Troncoso Gil, F. Trujillo Berraquero, M. A. Ulecia Martínez, J. Umaran Sánchez, C. Vaticón Herreros, A. Vázquez García, J. L. Vega Barbado, E. Velasco Espejo-Saavedra, T. Vicente Vera, M. Vida Gutiérrez, C. Villar Mariscal, G. Vives Boniato, L. Wu Amen, G. Yanes Bowden, J. C, Yañez Wonenburger, J. L. Zamorano Gómez, J. Zarauza Navarro; **Switzerland**: P. Monnier, A. Jaussi, A. Forclaz, M. Grobéty, L. Schlueter, C. Vuille, C. A. Nacht, D. Evéquoz, S. Ciaroni, F. Dominé, J. Bérubé, H. Rickli, J. Hellermann, R. Koller, G. Bourgeois, R. Engel, C. Niederberger, P. Stadler, M. Gnädinger, C. Schmied, T. Wettstein, P. Hilti, C. A. Chételat, F. Sepulcri, H. Brunner, J. Schindler, M. Kraus; **Thailand**: V. Vivekaphirat, S. Panpunnung, S. Kuanprasert, W. Wongcharoen, A. Phrommintikul, J. Harinasuta, O. Si, V. Chaithiraphan, T. Boonyasirinant,W. Boonyapisit, M. Kittipovanonth, A. Buakhamsri, D. Piyayotai, P. Hutayanon; **UK**: S. Junejo, O. Aiyegbayo, H. Ancliff, C. Bradshaw, R. Cervenak, H. Choi, E. George, I. Gilmour, D. Gough, A. Idrissi-Sbai, J. Ingham, B. Al-Khalidi, A. Liston, J. Mackrell, I. Pattison, R. Ramachandran, N. Ray, G. Reddy, I. Sen, K. Shetty, L. Singh, M. Stanley, A. Wallace, M.Weatherhead, T. Gilbert, G. McCansh, S. Higgins, C. Killeen, I. Cromarty, P. Franklin, E. Pinch, A. Dhesi, C. Dernedde, M. Lawrence, H. Simper, M. Noble, G. Dalton, L. Stevens, P. Berry, C. Hand, R. Oliver, H. Jones, P. Sampson, N. Taylor, R. Grogono, J. Dalrymple, A. Martin, S. Thurston, K. Elsby, M. Vallis, G. Morrison, C. Lang, A. Watson, A. Thomson, H. Dougall, B. La Hay, L. Compson, A. McCracken, J. Calder, F. Weber, D. Richmond, R. Brownlie, G. Brown, H. MacCowan, A. Heap, M. Perry, L. A. Holden, G. Scott, N. Haldane, S. Hood, I. Cullen, J. Bell P. McNaught, M. Sharif, J. Dunn, D. Hay, S. Ross, R. Shaw, L. Hay, S. Langridge, R. Burns, L. Crawford, A. Kennedy, D. Logan, P. McAlavey, M. Brown, P. Costello, G. McLaren, A. Potter, J. McPherson, M. Drijfhout, J. Finlayson, D. Troup, A. Woodall, J. Pearce, S. Williams, W. Parkar, A. Yusuf, I. Benett, P. Bishop, H. Thomas, I. Caldwell, P. Ormiston, S. Kwok, S. Wright, N. Kanumilli, P. Saul, H. Milligan, I. Wilkinson, A. Vance, N. Paul, C. Paul, I. Shaikh, R. Ellis, N. Vites, R. Steeds, D. Goodwin, A. Aftab, S. Banham, N. Chauhan,M. S. Grocutt, A. Gupte, R. Jordan, B. S. Jheeta, K. Ladha, M. Nazir, R. Pal, R. P. Patel, R. McManus, A. Singal, P. Saunders, A. B. Syed, A. Bahal, H. Dau, D. M. Walker, R. McNeilly, A. Bolidai, N. MacCarthy, D. Lawton, M. Vardhani, G. Sengupta, D. Kinloch, F. Howie, A. Serrano-Garcia, S. E. Paget, R. Till, P. Seal, J. Morrell, T. Maxwell, G. Singh, D. Warden, R. Elias, C. Dixon, R. K. Pandey, V. Challenor, S. Davies, M. Gibbs, A. Gillet, C. Goldie, I. Jarvis, P. Johnson, M. Malden, J. Moore, C. Morton, K. Nehrig, P. Sheringham, G. Wilson, J. Halcox, I. O'Connor, K. Ling, D. Edwards, H. Charles, A. Weatherup, E. Davies, N. Watkins, D. Morgan, R. Davies, A. Lindsay, D. Beacock, R. Balai, P. Kirmond, P. Brindle, C. Bundy, T. Cahill, A. Dayani, P. Eavis, S. Mohr, S. Hayne, C. Krasucki, M. Micheals, I. Orpen, I. Parker, R. Sewell, D. Sharp, A. Smith, A. Stevens, J. Upton, J. Victory, C Wernham, R. Davis, C. Mays, M. Andrews, J. Takhar, C. Travill, P. Choudhury, W. Matta, A. Ihonor, C. O'Dong, S. Rahman, P. Singer, S. Gillam, P. S. Bath, N. Razzaq, O. O'Toole, P. Rowe, H. Williams, P. Kalra, A. Allcock, A. Tucker, V. Sprott, K. Kyd, G. Cunliffe, C. Arden, A. Bateman, G. Kassianos, D. Sinclair, C. Turner, R. Jagathesan, F. Sattar, A. Ashford, A. Chukwu, H. Taylor, R. Pradhan, T. Rundell, R. Howlett, R. Bietzk, R. Patel, M. Myint, M. Partington, F. O'Reilly, M. Baverstock, S. Dixon,M. Tennekoon, N. Brand, P. Haimes, P. Keller, S.

Whetstone, R. Davis, C. Mays, M. Andrews, J. Takhar; L. Parker, M. Anscombe, G. Beale, L. Murphy, A. Smith, S. Brown, J. Lindford, A. Serrano, S. Preston, R. Sethi, S. Hutchinson; **Ukraine**: O. Kovyrshyna, V. Rogozhyna,T. Kiver, V. Vasylenko, L. Kucheryava, S. Salimova, V. Alekseenko, O. Gukov, I. Myhailiv, L. Kardashevskaya, O. Prikolota, O. Bashkirtcev, E. Andreev, L. Tkachenko, M. Mospan, V. Batushkin, D. Reshotko; L. Safonova, A. Ogorodnichuk, S. Pustovit, S. Romanov, L. Burlakova, Y. Voloshko, V. Lafarenko, Z. Vlasuk, O. Leshchuk, S. Chushak, V. Koval, O. Stasuk, O. Pogrebna, S. Kornienko, S. Tikhonova,T. Fesenko, T. Kuzmina, O. Ushakov, N. Vechtomova, L. Potapska, I. Illushechkin, E. Kryvenkova, O. Lysunets, O. Tsygankov, L. Bardachenko, L. Voloshyna, V. Ginzburg, L. Franskyavichene, T. Korotich, N. Vyshnevaya, N. Bilous, S. Kulinich, V. Kulik, I. Sadykova, O. Zalyzniak; T. Berezhna, S. Molotyagina; **Vietnam**: L. V. Nguyen, M. H. Pham, H. T. Pham, N. H. Khong, K. B. Do, T. B. LE, P. A. Do, T. C. Do, N. Q. Nguyen, Q. H. Do, K. C. Vu, N. H. Pham, T. H. T. Pham, M. C. Ta, D. P. Phan, T. T. H. Nguyen, T. T. N. Pham, T. L. To, V. T. Le, L. Dang, L. Bui, T. T. H. Pham, H. H. Phan, T. T. H. Bui, T. V. A. Tuong, T. P. Nguyen, T. H. Nguyen, B. K. Nguyen, D. B. Vu, N. S. Pham, T. Q. Do, T. S. Pham, V. D. Dang, D. T. Le, V. C. Do, T. K. L. Nguyen, H. D. Luong, T. Q. Luu, N. V. Pham, T. K. Huynh, N. T. H. Tu, K. A. Ngo, T. T. C. Nguyen, T. T. L. ONG, V. B. Doan, T. B. Kim, T. N. Vo, T. T. T. Tran, T. A. Nguyen, V. D. Tran, A. K. Nguyen, A. C. Tran, M. H. Ngo, N. H. Vu, I. T. Ly, N. P. H. Tran, L. U. P. Tran, T. N. Nguyen, T. H. Tran, P. H. Truong, T. L. Mai, V. S. Hoang, C. M. A. Bui, V. P. Dang, Q. B. Truong, M. P. Vo, V. T. Nguyen, N. H. Chau, T. T. H. Ta, H. N. Dinh, H. Tran, H. K. N. Nguyen; **West Indies**: A. Chung, E. Chung, B. Martina-Hooi, R. Angela, P. Ramoutar, R. Fillet, R. Tilluckdharry, T. Dookie, E. Foster, C. Hart, F. Omardeen, S. Ramphall, C. Lalla, R. Henry, J. Cheng, V. Elliott, H. Falconer, L. Hurlock-Clarke, R. Ishmael, G. Lalljie, K. Lee, A. Liqui-Lung, R. Massay, H. Mohammed, C. Brown, R. Daniel, M. Didier.

Table I. Countries in the Six Defined Geographic Areas

Region	Countries
West/central Europe	Austria<br>Belgium/Luxembourg<br>Bulgaria<br>Czech Republic<br>Denmark<br>France<br>Germany<br>Greece<br>Hungary<br>Ireland<br>Italy<br>Latvia<br>Lithuania<br>Netherlands<br>Poland<br>Portugal<br>Romania<br>Slovakia<br>Slovenia<br>Spain<br>Switzerland
Eastern Europe	Russia<br>Ukraine
Gulf countries	Bahrain<br>Kuwait<br>Oman<br>Qatar<br>Saudi Arabia<br>United Arab Emirates
Asia	Brunei<br>China<br>India<br>Korea<br>Malaysia<br>Singapore<br>Thailand<br>Vietnam
Central/South America	Argentina<br>Brazil<br>Mexico<br>West Indies
Commonwealth countries	Australia<br>Canada<br>South Africa<br>United Kingdom

Table II. Prevalence of Angina at Baseline According to Each of the Inclusion Criteria (Not Mutually Exclusive)

**Inclusion criterion**	**Prevalence of angina at baseline**
Documented myocardial infarction (>3 months ago) (n=18 869)	4423 (23.4)
Coronary angiography showing ≥1 coronary stenosis >50% (n=19 987)	3675 (18.4)
Chest pain with myocardial ischemia (n=7537)	2541 (33.7)
Percutaneous coronary intervention or coronary artery bypass graft (>3 months ago) (n=22 826)	3553 (15.6)
Variables are presented as count and percentage.	

Table III. 5-year Outcomes According to Anginal Status at Baseline

Outcome	Event rate, % (95% CI)		Adjusted HR (95% CI)*	*P* value
	Angina (n=7212)	No angina (n=25 479)		
Cardiovascular death or myocardial infarction	9.1 (8.4−9.8)	6.5 (6.2−6.9)	1.20 (1.08−1.33)	<0.001
Myocardial infarction	4.8 (4.3−5.3)	2.9 (2.7−3.1)	1.50 (1.30−1.74)	<0.001
Cardiovascular death	5.8 (5.2−6.3)	4.6 (4.4−4.9)	1.04 (0.92−1.19)	0.50
All-cause death	8.4 (7.8−9.1)	7.4 (7.1−7.8)	0.97 (0.87−1.08)	0.59
Elective revascularization	3.7 (3.2−4.1)	2.3 (2.1−2.5)	1.73 (1.46−2.05)	<0.001

Adjusted HRs and 95% CIs calculated using Cox proportional hazard model adjusted on the same variables as in the manuscript.
*Relative to patients without angina at baseline.
CI indicates confidence interval; HR, hazard ratio.

Table IV. Baseline Characteristics of Patients Alive With Missing Angina Status at 1 and 5 Years of Follow-Up

	**Available angina status at 1 year (n=30 558)**	**Missing angina status at 1 year (n=1722)**	***P* value**	**Available angina status at 5 year (n=21 927)**	**Missing angina status at 5 years (n=8220)**	***P* value**
Demographic characteristics						
Age, years	64.1 ± 10.4	64.9 ± 11.0	<0.001	63.3 ± 10.1	64.6 ± 10.9	<0.001
Female sex	6852 (22.4)	378 (22.1)	0.75	4992 (22.8)	1791 (21.8)	0.08
Body mass index, kg/m^2	27.9 ± 4.6	27.6 ± 4.6	0.09	28.0 ± 4.5	27.7 ± 4.6	<0.001
Cardiovascular risk factor						
Smoking status			0.007			0.78
Current	3783 (12.4)	241 (14.0)		2740 (12.5)	1011 (12.3)	
Former	14 158 (46.3)	734 (42.6)		10 071 (45.9)	3757 (45.7)	
Never	12 617 (41.3)	747 (43.4)		9116 (41.6)	3452 (42.0)	
Dyslipidemia	22935 (75.1)	1249 (72.6)	0.02	16 399 (74.8)	6219 (75.7)	0.11
Treated hypertension	21 700 (71.0)	1201 (69.8)	0.29	15 772 (71.9)	5524 (67.2)	<0.001
Family history of premature coronary artery disease	8718 (28.5)	501 (29.1)	0.63	6330 (28.9)	2379 (28.9)	0.90
Diabetes	8819 (28.9)	515 (29.9)	0.35	6103 (27.8)	2402 (29.2)	0.02
Physical activity			<0.001			<0.001
None	4809 (15.7)	355 (20.6)		3113 (14.2)	1492 (18.2)	
Light physical activity most weeks	15 712 (51.4)	889 (51.7)		11 581 (52.8)	3910 (47.6)	
>20 min physical activity once or twice weekly	5174 (16.9)	255 (14.8)		3745 (17.1)	1439 (17.5)	
>20 min physical activity ≥3 times weekly	4856 (15.9)	222 (12.9)		3487 (15.9)	1372 (16.7)	
Medical history						
Myocardial infarction	18 300 (59.9)	1008 (58.6)	0.30	13 158 (60.0)	4752 (57.8)	<0.001
Percutaneous coronary intervention	17 896 (58.6)	1062 (61.7)	0.01	12 741 (58.1)	5201 (63.3)	<0.001
Coronary artery bypass graft	7198 (23.6)	389 (22.6)	0.39	5063 (23.1)	1871 (22.8)	0.57
Cerebrovascular disease						
Stroke	1206 (3.9)	67 (3.9)	0.96	815 (3.7)	289 (3.5)	0.43
Transient ischemic attack	926 (3.0)	46 (2.7)	0.44	604 (2.8)	252 (3.1)	0.16
Carotid atheromatous disease	2310 (7.6)	109 (6.3)	0.07	1563 (7.1)	619 (7.5)	0.24
Peripheral artery disease						
Lower extremity artery disease	2988 (9.8)	176 (10.2)	0.57	1862 (8.5)	917 (11.2)	<0.001
Aortic abdominal aneurysm	459 (1.5)	33 (1.9)	0.20	236 (1.1)	176 (2.1)	<0.001
Hospitalization for heart failure	1365 (4.5)	96 (5.6)	0.03	807 (3.7)	390 (4.7)	<0.001
Atrial fibrillation/flutter	2113 (6.9)	134 (7.8)	0.18	1349 (6.2)	548 (6.7)	0.10

	**Available angina status at 1 year (n=30 558)**	**Missing angina status at 1 year (n=1722)**	***P* value**	**Available angina status at 5 year (n=21 927)**	**Missing angina status at 5 years (n=8220)**	***P* value**
Asthma/chronic obstructive pulmonary disease	2228 (7.3)	141 (8.2)	0.17	1455 (6.6)	612 (7.4)	0.01
Pacemaker	726 (2.4)	43 (2.5)	0.81	413 (1.9)	224 (2.7)	<0.001
Diagnosis of coronary artery disease						
Time since diagnosis, years	6.9 ± 6.3	6.4 ± 6.2	0.006	6.6 ± 6.0	7.0 ± 6.4	<0.001
Coronary angiography performed before inclusion	26 085 (85.4)	1512 (88.2)	0.001	18 535 (84.5)	7338 (89.4)	<0.001
Non-invasive test performed before inclusion	19 016 (62.2)	1143 (66.8)	<0.001	13 192 (60.2)	5725 (69.8)	<0.001
Evidence of myocardial ischemia at baseline	5003 (16.4)	224 (13.1)	<0.001	3607 (16.5)	1284 (15.6)	0.10
Angiographic findings	n=26 061	n=1493	0.06	n=18 526	n =7305	0.001
No significant stenosis	952 (3.7)	44 (2.9)		709 (3.8)	228 (3.1)	
One-vessel disease	10 710 (41.1)	654 (43.8)		7685 (41.5)	3173 (43.4)	
Multivessel disease	14 399 (55.3)	795 (53.2)		10 132 (54.7)	3904 (53.4)	
Coronary artery involved						
Left main	2694 (8.8)	120 (7.0)	0.01	1983 (9.0)	606 (7.4)	<0.001
Left anterior descending artery	17 817 (58.3)	1000 (58.3)	1.00	12 635 (57.6)	4956 (60.4)	0.10
Circumflex coronary artery	10 997 (36.0)	614 (35.8)	0.91	7746 (35.3)	2981 (36.3)	0.11
Right coronary artery	13 285 (43.5)	760 (44.3)	0.50	9355 (42.7)	3685 (44.9)	<0.001
Bypass graft	2444 (8.0)	146 (8.5)	0.47	1682 (7.7)	671 (8.2)	0.15
Left ventricular ejection fraction, %	56.2 ± 10.9	56.4 ± 11.8	0.46	56.2 ± 10.5	57.0 ± 11.3	<0.001
Symptoms						
Angina at baseline	6804 (22.2)	295 (17.1)	<0.001	5286 (24.1)	1306 (15.9)	<0.001
Canadian Cardiovascular Society class (if angina)			0.04			<0.001
I	1932 (28.4)	104 (35.3)		1482 (28.0)	436 (33.4)	
II	3639 (53.5)	141 (47.8)		2881 (54.5)	639 (48.9)	
III/IV	1233 (18.1)	50 (16.9)		923 (17.5)	231 (17.7)	
Heart failure symptoms	n=30557	n=1717	<0.001	n=21 927	n=8214	<0.001
No heart failure	25 907 (84.8)	1558 (90.7)		18 364 (83.8)	7480 (91.1)	
New York Heart Association class II	3911 (12.8)	128 (7.5)		3033 (13.8)	606 (7.4)	
New York Heart Association class III	739 (2.4)	31 (1.8)		530 (2.4)	128 (1.6)	
Pulse, bpm	68.2 ± 10.6	68.8 ± 10.8	0.02	68.3 ± 10.5	67.8 ± 10.7	<0.001
Systolic blood pressure, mmHg	131.1 ± 16.6	130.8 ± 16.9	0.56	130.8 ± 16.5	131.4 ± 16.6	0.01
Diastolic blood pressure, mmHg	77.3 ± 10.0	76.9 ± 10.0	0.15	77.5 ± 9.9	77.0 ± 9.7	<0.001

	**Available angina status at 1 year (n=30 558)**	**Missing angina status at 1 year (n=1722)**	***P* value**	**Available angina status at 5 year (n=21 927)**	**Missing angina status at 5 years (n=8220)**	***P* value**
Baseline medication						
Aspirin	26 843 (87.8)	1507 (87.9)	0.95	19 639 (89.6)	6993 (85.2)	<0.001
Thienopyridine	8253 (27.0)	514 (30.5)	0.002	5453 (24.9)	2805 (34.3)	<0.001
Dual antiplatelet therapy	8484 (27.8)	560 (32.6)	<0.001	6064 (27.7)	2511 (30.6)	<0.001
Oral anticoagulant	2459 (8.0)	142 (8.4)	0.65	1574 (7.2)	720 (8.8)	<0.001
Oral anticoagulant and antiplatelet drug	1564 (5.1)	92 (5.4)	0.71	1027 (4.7)	482 (5.9)	<0.001
Lipid-lowering drug	28 258 (92.5)	1568 (91.3)	0.09	20 280 (92.5)	7643 (93.0)	0.11
Statin	25 399 (83.1)	1363 (79.4)	<0.001	18 318 (83.5)	6710 (81.7)	<0.001
Beta-blocker	23 089 (75.6)	1223 (71.2)	<0.001	16 824 (76.7)	5916 (72.0)	<0.001
Ivabradine	3074 (10.1)	104 (6.1)	<0.001	2245 (10.2)	715 (8.7)	<0.001
Calcium channel blocker	8361 (27.4)	434 (25.3)	0.07	5952 (27.1)	2228 (27.1)	1.00
Long-acting nitrate	6654 (21.8)	369 (21.5)	0.82	4807 (21.9)	1555 (18.9)	<0.001
Other antianginal agent	4337 (14.2)	155 (9.1)	<0.001	3263 (14.9)	948 (11.6)	<0.001
Diuretic	8876 (29.0)	483 (28.2)	0.47	6059 (27.6)	2248 (27.4)	0.67
Angiotensin-converting enzyme inhibitor or angiotensin receptor blocker	23 307 (76.3)	1295 (75.7)	0.60	16 805 (76.6)	6135 (74.7)	<0.001
Insulin	1925 (6.3)	101 (5.9)	0.52	1259 (5.7)	510 (6.2)	0.13
Oral antidiabetes drug	6385 (20.9)	378 (22.0)	0.27	4419 (20.2)	1798 (21.9)	<0.001
Nonsteroidal anti-inflammatory drug	1504 (4.9)	91 (5.3)	0.49	1073 (4.9)	398 (4.9)	0.90
Thyroid hormone replacement therapy	1332 (4.4)	75 (4.4)	0.98	966 (4.4)	319 (3.9)	0.05
Proton pump inhibitor	7514 (24.6)	457 (26.7)	0.05	5097 (23.2)	2264 (27.6)	<0.001

Variables are presented as count and percentage. Includes patients with previous myocardial infarction or revascularization during follow-up.
Percentages are based on available data.

Table V. **Severity of Angina at Baseline, 1-Year and 5-Year Follow-Up, in 7212 Patients with Angina at Baseline**

**Severity**	**Baseline data**			**1-year data**	**5-year data**
	**Patients with angina at baseline (n=7212)**	**Patients with angina at baseline (but not at 1 year)* (n=2858)**	**Patients with angina at baseline and 1 year* (n=3660)**	**Patients with angina at 1 year* (n=4876)**	**Patients with angina at 5 years* (n=2682)**
CCS 1 or 2 angina	5897/7211 (81.8)	2530/2857 (88.5)	2817/3660 (77.0)	4117/4873 (84.4)	2285/2681 (85.2)
CCS 3 or 4 angina	1314/7211 (12.2)	327/2857 (11.4)	843/3660 (23.0)	756/4873 (15.5)	396/2681 (14.8)
Number of NTG puffs per month	5.7 ± 10.0 (n=7210)	3.5 ± 7.7 (n=2857)	7.5 ± 11.1 (n=3660)	5.4 ± 9.2 (n=4870)	4.2 ± 6.8 (n=2680)
Number of angina attacks per month	7.8 ± 10.1 (n=7211)	5.1 ± 7.9 (n=2858)	9.3 ± 11.1 (n=3660)	6.8 ± 9.3 (n=4872)	5.5 ± 7.2 (n=2680)

Dichotomous variables are reported as count with percentage. Continuous variables are reported as mean (SD).
*Patients were excluded at time of an event (death, myocardial infarction, or revascularization).
CCS indicates Canadian Cardiovascular Society; NTG, nitroglycerin.

Figure I. Graphic Representation of the Number of Patients Fulfilling Each of the Four Inclusion Criteria (Not Mutually Exclusive)

Abbreviation: MI, myocardial infarction.

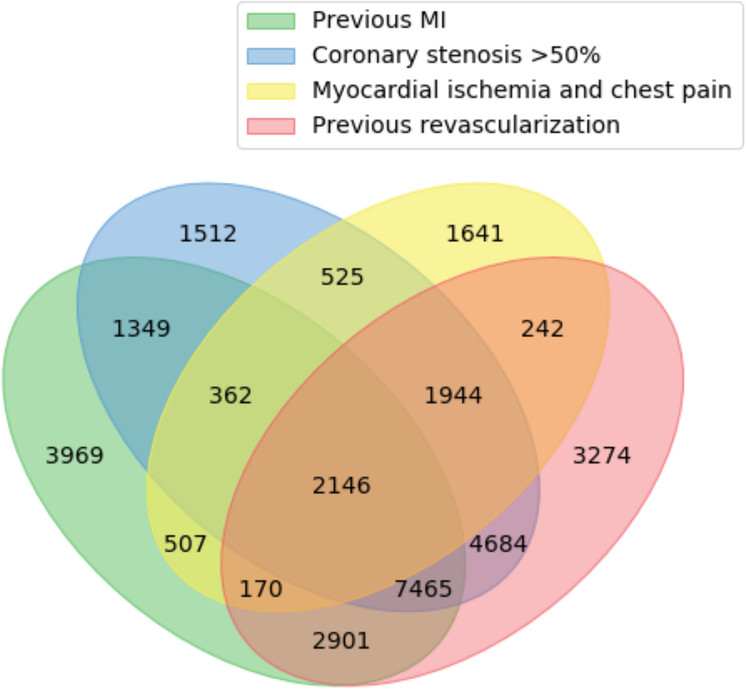

Figure II. Evolution of Angina in Patients With Stable Coronary Artery Disease Without Imputation of Missing Values

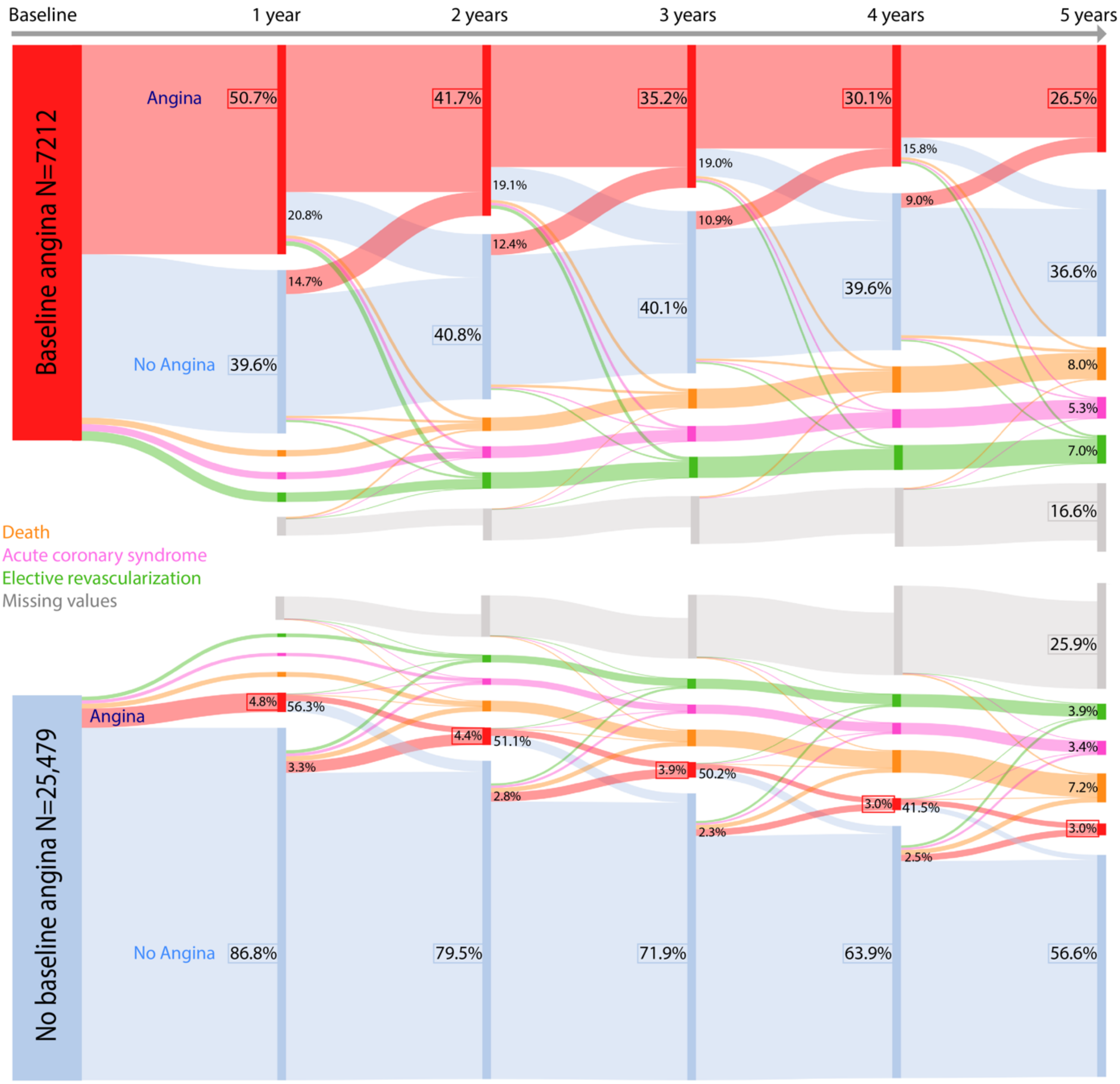

Missing values are in grey. Percentages on nodes reflect the proportion of patients compared to the initial group (baseline or no baseline angina). Percentages shown on the cross-over represent the proportion of patients according to the value of origin node.

Figure III. **Evolution of Angina in Patients with Stable Coronary Artery Disease: Full Population Scale**

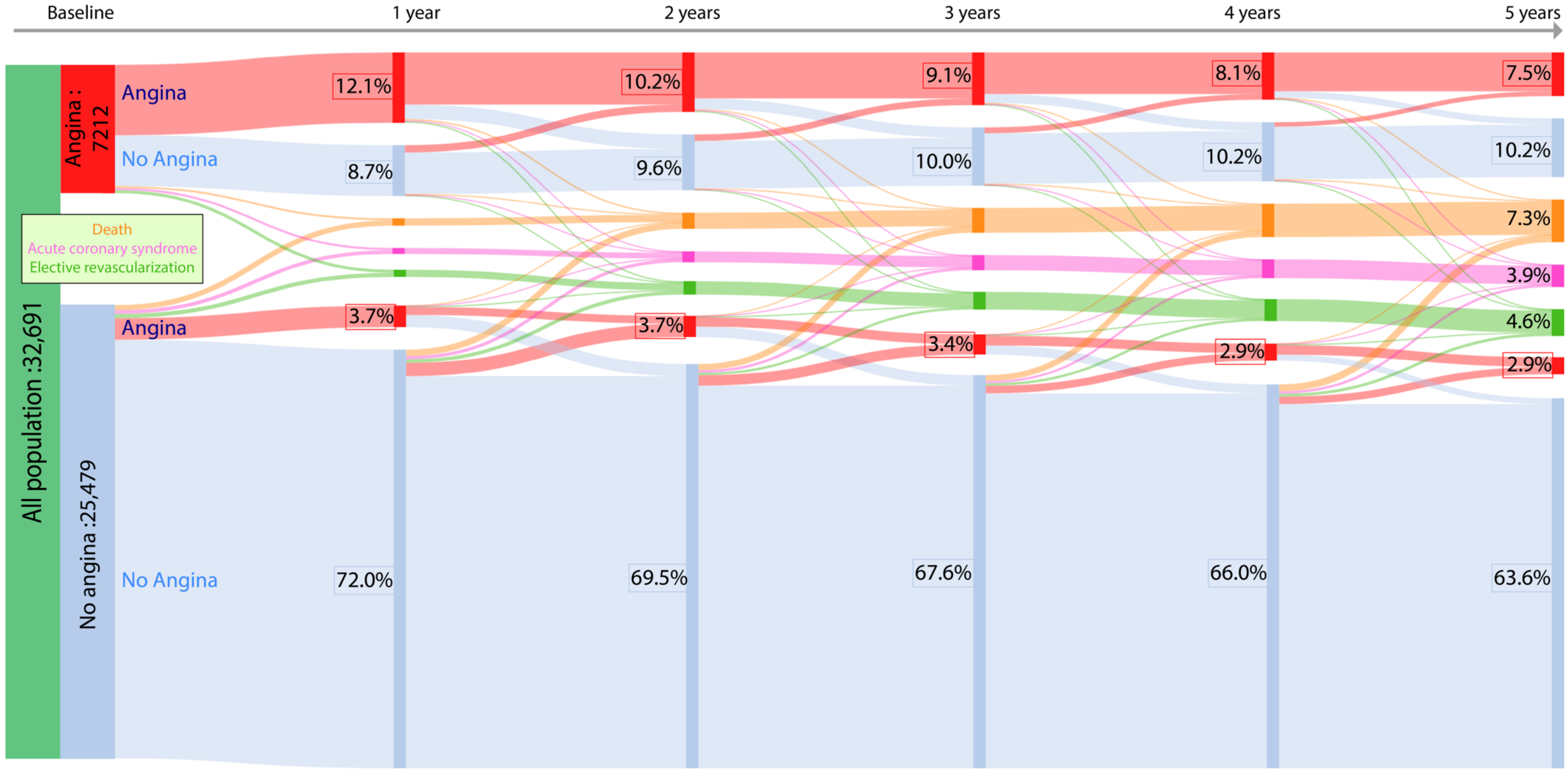

Figure IV. **Sensitivity Analysis: Exclusion of Patients From Eastern Europe; Evolution of Angina in Patients With Stable Coronary Artery Disease**

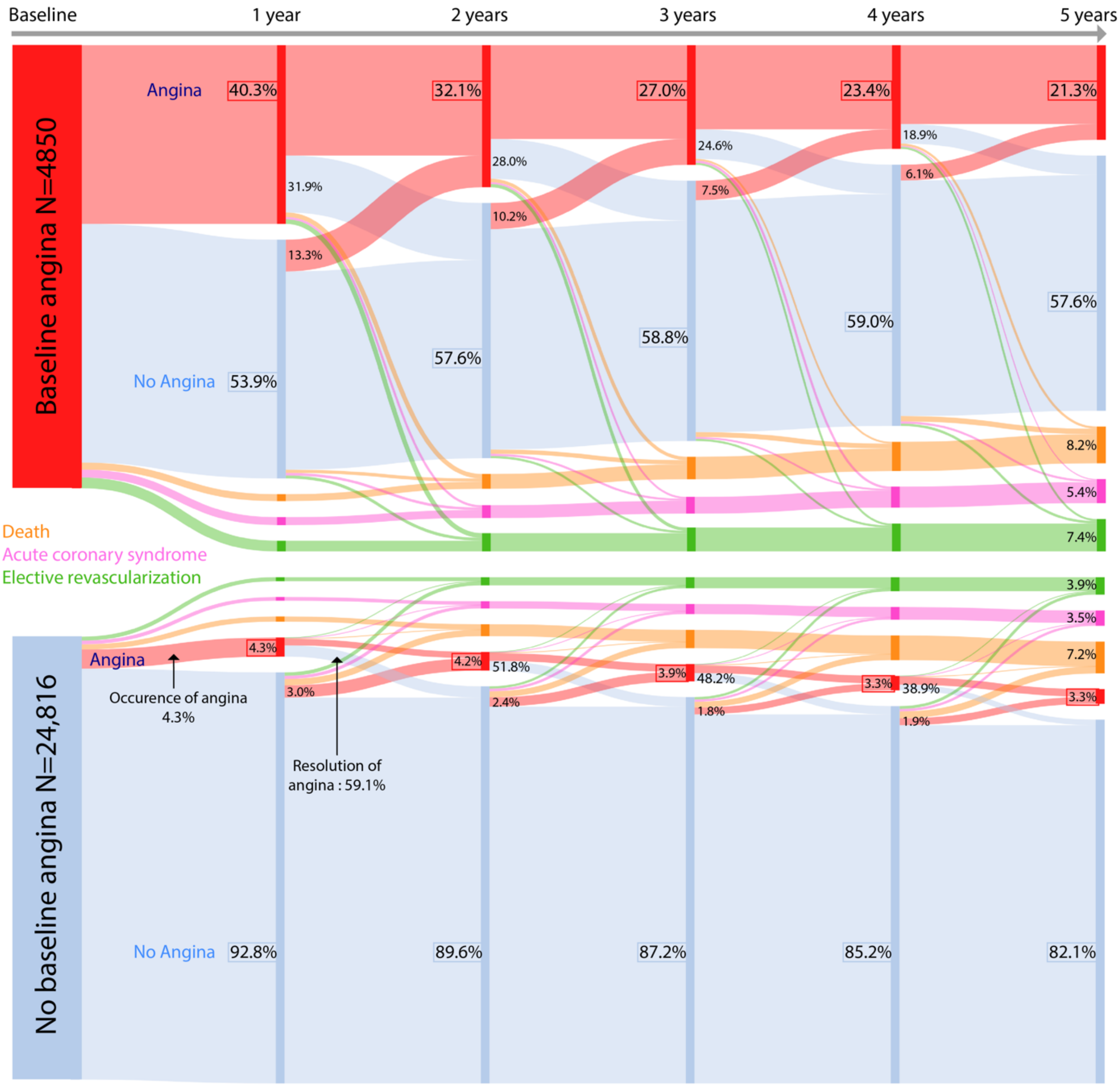

Figure V. Sensitivity Analysis: Exclusion of Patients From Eastern Europe; Clinical Outcomes According to the Evolution of Angina at 1 Year

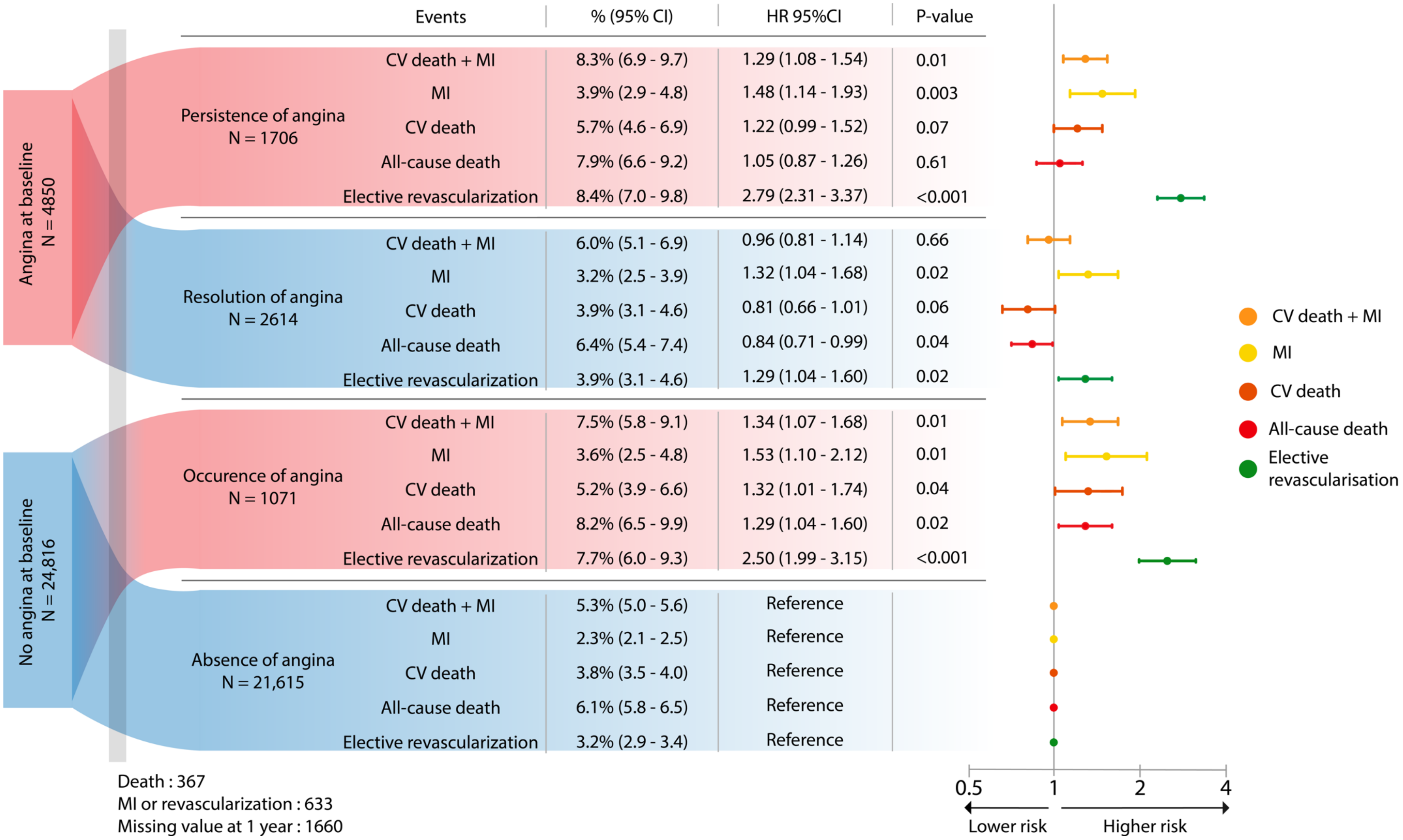

Group	Events	% (95% CI)	HR 95%CI	P-value
Persistence of angina N = 1706	CV death + MI	8.3% (6.9 - 9.7)	1.29 (1.08 - 1.54)	0.01
	MI	3.9% (2.9 - 4.8)	1.48 (1.14 - 1.93)	0.003
	CV death	5.7% (4.6 - 6.9)	1.22 (0.99 - 1.52)	0.07
	All-cause death	7.9% (6.6 - 9.2)	1.05 (0.87 - 1.26)	0.61
	Elective revascularization	8.4% (7.0 - 9.8)	2.79 (2.31 - 3.37)	<0.001
Resolution of angina N = 2614	CV death + MI	6.0% (5.1 - 6.9)	0.96 (0.81 - 1.14)	0.66
	MI	3.2% (2.5 - 3.9)	1.32 (1.04 - 1.68)	0.02
	CV death	3.9% (3.1 - 4.6)	0.81 (0.66 - 1.01)	0.06
	All-cause death	6.4% (5.4 - 7.4)	0.84 (0.71 - 0.99)	0.04
	Elective revascularization	3.9% (3.1 - 4.6)	1.29 (1.04 - 1.60)	0.02
Occurence of angina N = 1071	CV death + MI	7.5% (5.8 - 9.1)	1.34 (1.07 - 1.68)	0.01
	MI	3.6% (2.5 - 4.8)	1.53 (1.10 - 2.12)	0.01
	CV death	5.2% (3.9 - 6.6)	1.32 (1.01 - 1.74)	0.04
	All-cause death	8.2% (6.5 - 9.9)	1.29 (1.04 - 1.60)	0.02
	Elective revascularization	7.7% (6.0 - 9.3)	2.50 (1.99 - 3.15)	<0.001
Absence of angina N = 21,615	CV death + MI	5.3% (5.0 - 5.6)	Reference	
	MI	2.3% (2.1 - 2.5)	Reference	
	CV death	3.8% (3.5 - 4.0)	Reference	
	All-cause death	6.1% (5.8 - 6.5)	Reference	
	Elective revascularization	3.2% (2.9 - 3.4)	Reference	

Figure VI. Consequences of Angina and Evolution of Angina at 1 Year (Primary Outcome of Cardiovascular Death or Myocardial Infarction) in Subgroups

		Number of patients	CV death + MI HR (95% CI)	P-value
**Patients with prior MI**				
Angina at baseline*		19402	1.30 (1.14-1.47)	<0.001
At 1 year	Persistence of angina	2494	1.43 (1.18-1.73)	<0.001
	Regression of angina	1581	1.07 (0.88-1.30)	0.52
	Occurence of angina	716	1.36 (1.04-1.77)	0.02
**Patients without prior MI**				
Angina at baseline*		12974	1.06 (0.87-1.26)	0.58
At 1 year	Persistence of angina	1166	1.14 (0.83-1.56)	0.43
	Regression of angina	1277	0.86 (0.63-1.16)	0.32
	Occurence of angina	500	1.45 (1.00-2.09)	0.05
**Patients with myocardial ischemia at baseline**				
Angina at baseline*		5259	1.13 (0.91-1.41)	0.28
At 1 year	Persistence of angina	1067	1.28 (0.93-1.75)	0.13
	Regression of angina	901	0.74 (0.52-1.05)	0.09
	Occurence of angina	182	1.34 (0.79-2.29)	0.28
**Patients with diabetes at baseline**				
Angina at baseline*		9393	1.25 (1.06-1.48)	0.008
At 1 year	Persistence of angina	936	1.27 (0.97-1.66)	0.08
	Regression of angina	911	1.22 (0.96-1.55)	0.11
	Occurence of angina	366	1.28 (0.89-1.84)	0.18
**Patients with multivessel disease**				
Angina at baseline		15283	1.25 (1.09-1.44)	0.002
At 1 year	Persistence of angina	1301	1.38 (1.11-1.71)	0.004
	Regression of angina	1245	0.95 (0.75-1.19)	0.64
	Occurence of angina	601	1.50 (1.13-2.00)	0.004

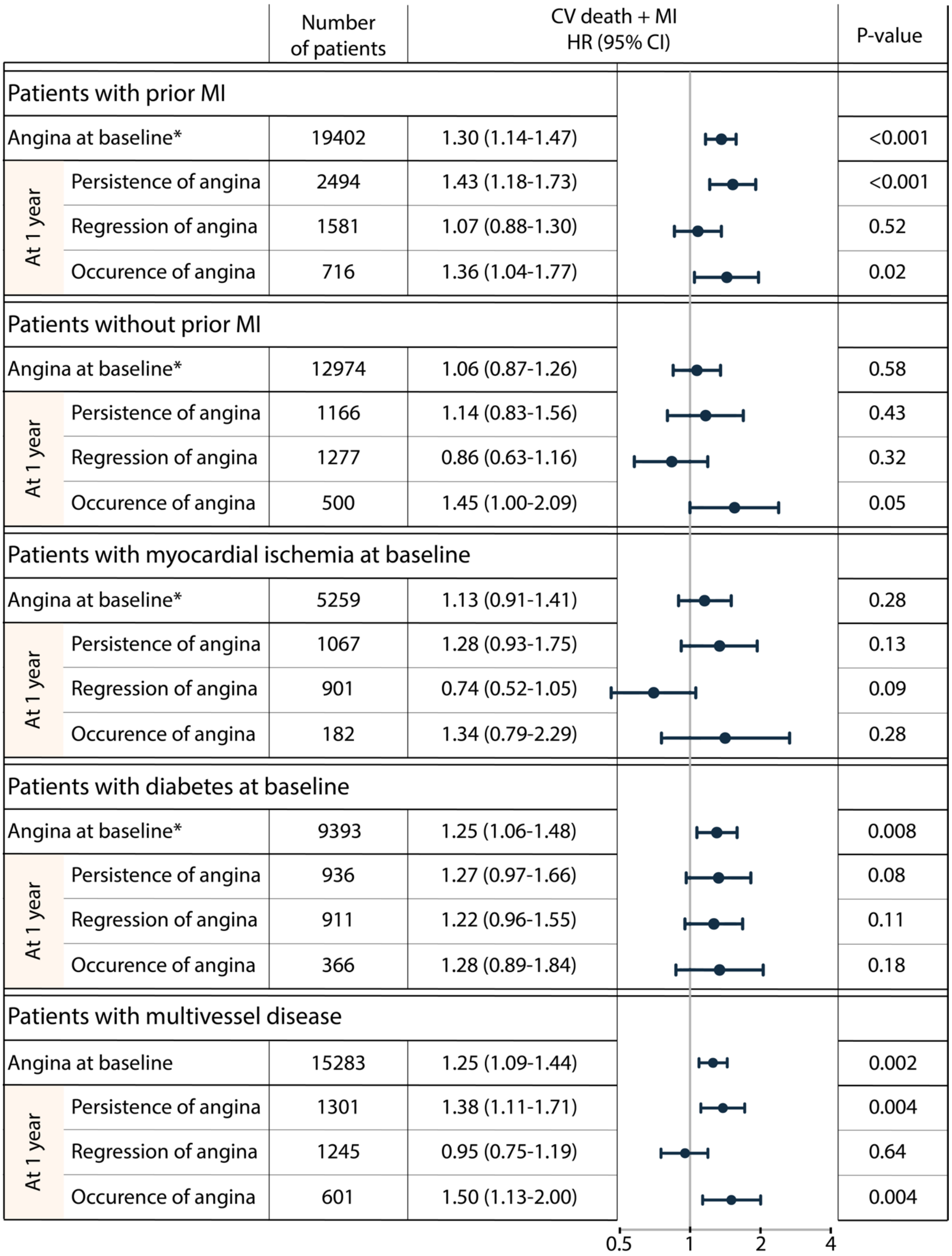

All HRs are adjusted and estimated from Cox proportional hazards model.
*HR relative to patients who did not experience angina at baseline. Patients who did not experience angina either at baseline or 1 year were used as the reference to estimate HR according to the evolution of angina at 1 year.
Abbreviations: CI, confidence interval; CV, cardiovascular; HR, hazard ratio; MI, myocardial infarction.
