# Supplementary material for: International Observational Analysis of Evolution and Outcomes of Chronic Stable Angina: The Multinational CLARIFY Study
Source: Circulation. 2021 Jul 15;144(7):512–23. doi: 10.1161/CIRCULATIONAHA.121.054567 (PMC8360663; doi:10.1161/CIRCULATIONAHA.121.054567)
Supplement: Supplementary file 2 [file cir-144-512-s002.pdf]

OXFORD UNIVERSITY PRESS LICENSE TERMS AND CONDITIONS

Jul 08, 2021

This Agreement between Hôpital Bichat, Université de Paris -- Ph Gabriel Steg ("You") and Oxford University Press ("Oxford University Press") consists of your license details and the terms and conditions provided by Oxford University Press and Copyright Clearance Center.

The publisher has provided special terms related to this request that can be found at the end of the Publisher's Terms and Conditions.

All payments must be made in full to CCC. For payment instructions, please see information listed at the bottom of this form.

License Number	5080861339463
License date	Jun 02, 2021
Licensed Content Publisher	Oxford University Press
Licensed Content Publication	European Heart Journal
Licensed Content Title	Long-term outcomes of chronic coronary syndrome worldwide: insights from the international CLARIFY registry
Licensed Content Author	Sorbets, Emmanuel; Fox, Kim M
Licensed Content Date	Sep 3, 2019
Licensed Content Volume	41
Licensed Content Issue	3
Type of Use	Journal
Requestor type	Author of this OUP content
Pharmaceutical support or sponsorship for this project	Yes
Format	Print and electronic
Portion	Figure/table
Number of figures/tables	1
Will you be translating?	No
Circulation/distribution	10000
Title of new article	International Observational Analysis of Evolution and Outcomes of Chronic Stable Angina The Multinational Observational CLARIFY Study
Lead author	Jules Mesnier
Title of targeted journal	Circulation
Publisher	American Heart Assocation
Expected publication date	Aug 2021
Portions	Half of Table 1: Baseline characteristics according to history of MI status or according to current angina status
Requestor Location	Hôpital Bichat, Université de Paris<br>46 Rue Henri Huchard<br><br>Paris, 75018<br>France<br>Attn: Hôpital Bichat, Université de Paris
Publisher Tax ID	GB125506730
Billing Type	Invoice
Billing Address	Hôpital Bichat, Université de Paris

35 rue de Verdun
92284 SURESNES Cedex

Paris, France 92284
Attn: Hôpital Bichat, Université de Paris

Customer reference info	VAT : FR 01 602 048 506
Total	**2,490.45 EUR**
Terms and Conditions	

STANDARD TERMS AND CONDITIONS FOR REPRODUCTION OF MATERIAL FROM AN OXFORD UNIVERSITY PRESS JOURNAL

1. Use of the material is restricted to the type of use specified in your order details.
2. This permission covers the use of the material in the English language in the following territory: world. If you have requested additional permission to translate this material, the terms and conditions of this reuse will be set out in clause 12.
3. This permission is limited to the particular use authorized in (1) above and does not allow you to sanction its use elsewhere in any other format other than specified above, nor does it apply to quotations, images, artistic works etc that have been reproduced from other sources which may be part of the material to be used.
4. No alteration, omission or addition is made to the material without our written consent. Permission must be re-cleared with Oxford University Press if/when you decide to reprint.
5. The following credit line appears wherever the material is used: author, title, journal, year, volume, issue number, pagination, by permission of Oxford University Press or the sponsoring society if the journal is a society journal. Where a journal is being published on behalf of a learned society, the details of that society must be included in the credit line.
6. For the reproduction of a full article from an Oxford University Press journal for whatever purpose, the corresponding author of the material concerned should be informed of the proposed use. Contact details for the corresponding authors of all Oxford University Press journal contact can be found alongside either the abstract or full text of the article concerned, accessible from www.oxfordjournals.org Should there be a problem clearing these rights, please contact journals.permissions@oup.com
7. If the credit line or acknowledgement in our publication indicates that any of the figures, images or photos was reproduced, drawn or modified from an earlier source it will be necessary for you to clear this permission with the original publisher as well. If this permission has not been obtained, please note that this material cannot be included in your publication/photocopies.
8. While you may exercise the rights licensed immediately upon issuance of the license at the end of the licensing process for the transaction, provided that you have disclosed complete and accurate details of your proposed use, no license is finally effective unless and until full payment is received from you (either by Oxford University Press or by Copyright Clearance Center (CCC)) as provided in CCC's Billing and Payment terms and conditions. If full payment is not received on a timely basis, then any license preliminarily granted shall be deemed automatically revoked and shall be void as if never granted. Further, in the event that you breach any of these terms and conditions or any of CCC's Billing and Payment terms and conditions, the license is automatically revoked and shall be void as if never granted. Use of materials as described in a revoked license, as well as any use of the materials beyond the scope of an unrevoked license, may constitute copyright infringement and Oxford University Press reserves the right to take any and all action to protect its copyright in the materials.
9. This license is personal to you and may not be sublicensed, assigned or transferred by you to any other person without Oxford University Press's written permission.
10. Oxford University Press reserves all rights not specifically granted in the combination of (i) the license details provided by you and accepted in the course of this licensing transaction, (ii) these terms and conditions and (iii) CCC's Billing and Payment terms and conditions.
11. You hereby indemnify and agree to hold harmless Oxford University Press and CCC, and their respective officers, directors, employs and agents, from and against any and all claims arising out of your use of the licensed material other than as specifically authorized pursuant to this license.
12. Other Terms and Conditions: -The co-authors on both papers are Roberto Ferrari, Ian Ford, Jean-Claude Tardif, Michal Tendera, Kim M Fox, and Philippe Gabriel Steg. Servier sponsored the CLARIFY registry. No sponsor funding to cover permission fees for the table, which is only a small part of the original and comprises data from the authors' own study. -Inclusion under a Creative Commons license or any other Open Access license allowing onward reuse is prohibited. -The credit line and any copyright/ disclaimer notices provided by OUP shall be included on the same page as the Material: Author, title, journal, year, volume, issue number, pagination, DOI number. Adapted and reproduced by permission of Oxford University Press on behalf of the European Society of Cardiology.

v1.4

You will be invoiced within 48 hours of this transaction date. You may pay your invoice by credit card upon receipt of the invoice for this transaction. Please follow instructions provided at that time.
To pay for this transaction now; please remit a copy of this document along with your payment. Payment should be in the form of a check or money order referencing your account number and this invoice number RLNK504101561.

Make payments to "COPYRIGHT CLEARANCE CENTER" and send to:

Copyright Clearance Center
29118 Network Place

Chicago, IL 60673-1291
Please disregard electronic and mailed copies if you remit payment in advance

Questions? customercare@copyright.com or +1-855-239-3415 (toll free in the US) or +1-978-646-2777.
